# Preclinical Study on Sabin Strain-Based DTaP-sIPV/Hib Pentavalent Vaccine: Evaluation of Repeated-Dose Toxicity and Immunogenicity

**DOI:** 10.3390/vaccines14010029

**Published:** 2025-12-25

**Authors:** Ting Zhao, Han Chu, Yan Ma, Qin Gu, Na Gao, Jingyan Li, Qiuyan Ji, Jiana Wen, Xiaoyu Wang, Guoyang Liao, Shengjie Ouyang, Wenzhu Hu, Hongwei Liao, Guang Ji, Hongbo Chen, Lujie Yang, Mingqing Wang, Ling Ping, Yuting Fu, Yixian Fu, Wenlu Kong, Huimei Zheng, Xinhua Qin, Lukui Cai, Jiangli Liang, Jingsi Yang

**Affiliations:** 1 Institute of Medical Biology, Chinese Academy of Medical Sciences & Peking Union Medical College, Kunming 650118, China; 2 State Key Laboratory of Respiratory Health and Multimorbidity, Beijing 100005, China; 3 Kunming Scientific and Technological Innovation Center for R&D and Industrialization of Vaccines Against Novel and Emerging Highly Pathogenic Pathogens, Kunming 650118, China

**Keywords:** Sabin inactivated poliovirus vaccine (Sabin IPV), DTacP-sIPV/Hib pentavalent vaccine, repeated-dose toxicity

## Abstract

Background: Pertussis, diphtheria, tetanus, poliomyelitis, and *Haemophilus influenzae* type b (Hib) infections pose severe threats to children’s health globally. This study evaluated the safety and immunogenicity of a novel Sabin strain-based adsorbed pentavalent vaccine (DTacP-sIPV/Hib), which offers potential advantages in biosafety and cost-effectiveness compared to wild-type poliovirus-based vaccines. Methods: A repeated-dose toxicity study was conducted in 190 Sprague-Dawley rats, randomly divided into negative control, adjuvant control, low-dose, and high-dose groups. Animals received five intramuscular injections at 21-day intervals, followed by a 56-day recovery period. Parameters assessed included local reactions, body temperature, hematology, serum biochemistry, coagulation, histopathology, T-cell subsets, cytokine levels, and antigen-specific immunogenicity. Results: The primary adverse reaction was dose-dependent local muscle swelling, which was fully reversible within 3–21 days. Only transient body temperature fluctuations and adjuvant-related hematological/biochemical abnormalities were observed, all resolving after the recovery period. No vaccine-related damage occurred in hepatic/renal function or immune organs. Immunogenicity data showed 100% seroconversion for all bacterial components 21 days after the first dose. The high-dose group achieved 100% seropositivity for all poliovirus serotypes after the second dose, while the low-dose group reached the same after the third dose, with no significant difference in antibody levels between dose groups. Conclusions: The DTacP-sIPV/Hib vaccine exhibits a favorable safety profile and robust immunogenicity in rats, supporting its further clinical development. The use of Sabin strains reduces biosafety risks and manufacturing costs, making this vaccine a promising candidate for immunization programs, especially in resource-limited regions.

## 1. Introduction

Pertussis, diphtheria, tetanus, poliomyelitis, and invasive diseases caused by *Haemophilus influenzae* type b (Hib) remain critical global public health threats to children’s lives [1,2]. The World Health Organization estimates that there are approximately 24.1 million cases of pertussis annually, resulting in 160,700 deaths among children under 5 years old [3]. Regarding diphtheria, due to disruptions in vaccination efforts during the COVID-19 pandemic, globally reported cases surged from 2022 to 2023, with outbreaks in multiple countries and confirmed cases exceeding 18,000 [4]. Progress has been made in the prevention and control of tetanus, but challenges remain. In low-income countries, the risk of death for unvaccinated individuals can be as high as 50%; it is reported that over 30,000 people die from it annually [5]. More notably, the global situation of poliomyelitis prevention and control remains challenging. In 2024, 68 cases of wild poliovirus type 1 (WPV1) were reported, and the transmission of circulating vaccine-derived poliovirus (cVDPV) continues in multiple countries [6]. Hib is a significant pathogen of bacterial meningitis, and in areas with weak healthcare resources, its case fatality rate can remain high [7]. Vaccination, as a core means of preventing such diseases, is crucial for the efficiency of disease prevention and control.

Pentavalent vaccines (DTacP-IPV-Hib), combining diphtheria–tetanus–acellular pertussis (DTacP), inactivated poliovirus vaccine (IPV), and Hib vaccine, offer the advantage of “preventing five diseases with a single injection.” This significantly simplifies immunization schedules, reduces costs, and improves coverage and compliance [8]. The globally leading pentavalent vaccine, Sanofi’s Pentaxim, has been applied in over 100 countries and regions worldwide. By 2011, its cumulative administration exceeded 100 million doses, demonstrating favorable safety and efficacy [9,10,11,12]. However, it faces critical limitations: conventional DTacP-IPV-Hib pentavalent vaccines predominantly use wild-type strain-based inactivated poliovirus [11], which requires Biosafety Level 3 (BSL-3) production facilities [13,14]. This complex, high-cost process carries inherent biosafety risks from potential wild-type poliovirus leakage, directly contributing to low vaccination coverage in low- and middle-income countries (LMICs).

The pentavalent vaccine used in this study was independently developed with optimized core technologies to address the aforementioned issues, thereby further enhancing its safety. The vaccine employs the attenuated poliovirus Sabin strain instead of the wild strain for production, which lowers the required biosafety level for production facilities, eliminates the risk of wild virus leakage, and reduces production costs compared to using the wild strain [13,14].

Nevertheless, potential risks of antigen interference, immune interference, and synergistic adjuvant toxicity in multivalent vaccines—particularly cumulative toxicity to vital organs like the liver and kidneys—require systematic evaluation.

To address these concerns, this repeated-dose toxicity study in rats strictly simulated clinical immunization protocols. We comprehensively assessed impacts on hepatic/renal metabolism, hematopoietic function, and immune homeostasis through hematological parameters, serum biochemistry, and histopathology of immune organs. These preclinical safety data will provide critical risk indicators for subsequent human trials.

## 2. Materials and Methods

### 2.1. Vaccine

The pentavalent vaccine (DTacP-sIPV/Hib) used in this safety trial was developed and prepared by the Institute of Medical Biology, Chinese Academy of Medical Sciences(Kunming, Yunnan, China). It adopts a formulation combining a lyophilized *Haemophilus influenzae* type b (Hib) conjugate vaccine (1 vial/dose) with a liquid quadrivalent vaccine (Adsorbed acellular Pertussis-Sabin strain Inactivated Poliomyelitis Combined Vaccine, 0.5 mL/dose). Each 0.5 mL dose contains 12.5 Lf diphtheria toxoid, 3.5 Lf tetanus toxoid, acellular pertussis antigens [25 μg detoxified pertussis toxin (PT), 25 μg filamentous hemagglutinin (FHA), 8 μg pertactin (PRN)], inactivated Sabin polioviruses [30 D-antigen units (DU) Type 1, 32 DU Type 2, 45 DU Type 3] and 10 μg PRP of Hib covalently bound to tetanus toxoid (PRP-T). Other ingredients per 0.5 mL dose include 0.725 mg aluminum hydroxide as the adjuvant.

The DTacP-sIPV/Hib vaccine used in this safety evaluation study had undergone prior potency testing in NIH mice for its diphtheria, tetanus, pertussis, and Hib components prior to administration to SD rats. The in vivo potency of the diphtheria, tetanus, pertussis, and Hib vaccines met the standards specified in the Chinese Pharmacopoeia (Volume III, 2020 edition) for relevant vaccines. This includes minimum antibody potency requirements per dose in NIH mice: not less than 60 IU/mL for diphtheria, not less than 80 IU/mL for tetanus, not less than 8.0 IU/mL for pertussis, and a positivity rate exceeding 80% for Hib. Additionally, potency tests in Wistar rats demonstrated that the median effective dose (ED_50_) for each poliovirus type was not significantly inferior to that of the reference vaccine.

Prior to administration, the entire liquid vaccine component (0.5 mL) was injected into the lyophilized Hib component and mixed thoroughly. Additionally, aluminum hydroxide (adjuvant control) and 0.9% sodium chloride injection (negative control) were also prepared by the Institute of Medical Biology, Chinese Academy of Medical Sciences.

### 2.2. Test Animals

Specific Pathogen-Free (SPF) grade Sprague-Dawley (SD) rats were bred by Beijing Vital River Laboratory Animal Technology Co., Ltd. (Beijing, China). Production License Number and Issuing Authority: SCXK (Jing) 2021-0006 (Beijing Municipal Science and Technology Commission). Laboratory Animal Quality Certificate Numbers: No. 110011241104972046, No. 110011241104972141. A total of 190 rats were used: 95 males and 95 females. The SD rats were approximately 5–7 weeks old upon arrival at the laboratory, with body weights ranging from 144.2 to 182.6 g for males and 139.3–173.5 g for females. After a 3-day quarantine period and a 5-day acclimation period, the rats received their first injection at the age of 6–8 weeks. At this time, the experimental animals’ body weights ranged from 208.2 to 243.9 g for males and 172.4–207.8 g for females. All rats were housed in a barrier system environment with temperature controlled at 20–26 °C and humidity at 40–70%. Animals had free access to water and food. Prior to the formal experiment, a quarantine period of 3 days and an acclimation period of 5 days were conducted. Quarantine examinations included assessment of appearance, body shape, activity, respiration, coat, nose, oral cavity, eyes, ears, genitals, urine, and feces. This experiment was approved by the Institutional Animal Care and Use Committee (IACUC) of Shandong Xinbo Pharmaceutical Research Co., Ltd. (Jinan, Shangdong, China). (Approval No.: XB-IACUC-20241097).

### 2.3. Experimental Grouping

A total of 190 SD rats were randomly divided into four main test groups (Negative Control group, Adjuvant Control group, Low-Dose group, and High-Dose group) and three satellite groups using TOXSTAT2006 1.0 software based on sex and body weight. Main test groups: Each group contained 20 males and 20 females. Satellite groups: Each group contained 5 males and 5 females.

### 2.4. Administration Method

**Figure 1:**
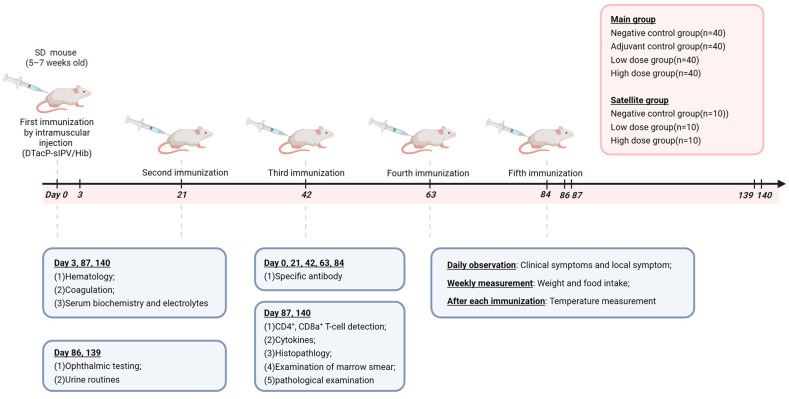
A schematic depicting the experimental process. 190 SD rats were randomly divided into four main test groups (negative control group, adjuvant control group, low-dose group, and high-dose group) and three satellite groups. Each group received one administration on days 0, 21, 42, 63, and 84, totaling five administrations. A 56-day recovery period followed the last administration. After each administration, the injection sites were thoroughly observed and palpated daily. Body weight and food intake were measured once per week for the main group. Serum biochemistry, pathology, hematology, T cells, neutralizing antibodies, cytokines, etc., were detected according to the study design.

The dosing regimen comprised intramuscular injections (0.25 mL per site) administered alternately in the medial thigh muscles of hind limbs using a 1 mL syringe. The requirement for the inoculation dose in long-term toxicity studies, as stated in the General Principles for Technical Review of Non-clinical Safety Evaluation of Preventive Biological Products, is that “the inoculation dose should, in principle, achieve the optimal immune response in the animal body. The dose that induces the optimal immune response in animals can be selected through immunogenicity studies and used for the long-term toxicity study; alternatively, the high dose intended for clinical use (calculated per human dose) can be directly applied in the long-term toxicity study.” To fully expose potential toxicity, the high-dose group received administrations via multiple bilateral sites in the hind limbs, with 0.25 mL per site, resulting in a cumulative administration volume of 0.5 mL per animal. The administration volume for the low-dose group was 0.25 mL per animal. The administered doses for the low- and high-dose groups corresponded to 0.5 and 1 times the clinical dose, respectively. Main study groups received: negative control (0.9% sodium chloride, 0.5 mL), adjuvant control (aluminum hydroxide, 0.5 mL), low-dose (half-dose pentavalent vaccine, 0.25 mL), and high-dose (full-dose pentavalent vaccine, 0.5 mL). Satellite groups mirrored this design with negative control, low-dose, and high-dose subgroups. All groups underwent five administrations on days 0, 21, 42, 63, and 84, followed by a 56-day recovery period post final dose (Figure 1).

### 2.5. Observations and Measurements

During dosing days, animals were observed three times daily (pre-dose in the morning, 30–60 min post dose, and in the afternoon), with daily post-dose detailed examination and palpation of the injection site. For non-dosing days, observations were conducted twice daily (morning and afternoon). Physiological monitoring included weekly body weight and 24 h food consumption measurements, alongside body temperature recordings at 4–6 h post dose, 24 h post dose, and 3 days post dose. Necropsy protocols for main study groups specified: 5 females and 5 males euthanized on day 3 post first dose for hematology, serum biochemistry, and coagulation analyses; 20 animals (10♀ + 10♂) and 10 animals (5♀ + 5♂) euthanized on day 3 and day 56 post last dose, respectively, for comprehensive testing (hematology/serum biochemistry/coagulation, CD4^+^/CD8a^+^ T cells, cytokines, organ wet weights, and histopathology). Pre-necropsy procedures on day 2 and day 55 post last dose included urinalysis and ophthalmic examinations. Satellite group animals underwent specific antibody detection pre dose and on day 21 post each dose. All procedures adhered to GLP standards with terminology aligned to ICH E3 and OECD guidelines.

Temperature Measurement: Rectal temperature was measured for all surviving animals in the main experimental group using a veterinary thermometer at 4–6 h after each administration, on the day following administration, and 3 days after administration.

Blood Analysis: On day 3 after the first administration, day 3 after the last administration, and day 56 after the last administration, animals scheduled for dissection were fasted overnight. They were anesthetized via subcutaneous injection in the neck/back region with a combination of tiletamine hydrochloride and zolazepam hydrochloride (30 mg/kg, 50 mg/mL, 0.6 mL/kg). Approximately 0.5 mL of blood was collected from the abdominal vena cava using EDTA anticoagulant. Hematological parameters were analyzed using the XN-1000V (BI) fully automated modular animal blood and body fluid analyzer.

Serum Biochemical Analysis: On day 3 after the first administration, day 3 after the last administration, and day 56 after the last administration, animals scheduled for dissection were fasted overnight. They were anesthetized as described above. Approximately 3 mL of blood was collected from the abdominal vena cava into plastic tubes without anticoagulant. The samples were left at room temperature and then centrifuged at 3000 rpm for 10 min to separate serum for biochemical testing. Serum biochemical parameters were analyzed using the Dimension RxL Max (Siemens Healthcare Diagnostics Inc., Brookfield, NY, USA) fully automated biochemical analyzer.

Coagulation Function Tests: On day 3 after the first administration, day 3 after the last administration, and day 56 after the last administration, animals scheduled for dissection were fasted overnight and anesthetized as described above. Approximately 1 mL of blood was collected from the abdominal vena cava using a 9:1 ratio of sodium citrate anticoagulant. The samples were centrifuged at 3000 rpm for 10 min to obtain plasma. Coagulation function was assessed using the ACL TOP 300 CTS fully automated coagulation analyzer (Werfen, Bedford, NY, USA) with the clotting method.

CD4^+^, CD8a^+^, and CD3^+^ T-cell Detection: On day 3 and day 56 after the last administration, animals scheduled for dissection were fasted overnight and anesthetized as described above. Approximately 0.6 mL of blood was collected from the abdominal vena cava into heparinized anticoagulant tubes and mixed thoroughly. About 30 μL of whole blood was used to prepare samples for flow cytometry analysis; the remaining blood was used for cytokine measurement. Using a flow cytometer, a specific number of cells were collected. The counts of CD3^+^CD4^+^ cells and CD3^+^CD8a^+^ cells were determined. The proportion of CD3^+^CD4^+^ cells and CD3^+^CD8a^+^ cells within the lymphocyte population was analyzed using guavasoft 1.0 software, and their ratio was calculated.

Cytokine Detection: Using the aliquoted whole blood from the CD4^+^/CD8a^+^ T-cell detection, samples were centrifuged at 3000 rpm for 10 min. The supernatant was collected and frozen for subsequent detection of the cytokines IFN-γ, TNF-α, and IL-2 using the CBA (Cytometric Bead Array) method.

Organ Weighing: On day 3 and day 56 after the last administration, animals scheduled for dissection were fasted overnight, anesthetized as described above, exsanguinated, and dissected. Gross observations were made during dissection. The wet weights of the brain, heart, liver, kidneys, adrenal glands, thymus, spleen, lungs, testes, epididymides, ovaries, and uterus were measured. The organ coefficient (organ wet weight in grams/body weight × 100 g) was calculated.

Specific Antibody Detection: Blood samples (approximately 0.6 mL) were collected from the jugular venous sinus of satellite group rats before the first administration and 21 days after each administration. The blood was placed into plastic tubes without anticoagulant, allowed to stand at room temperature, and then centrifuged at 3000 rpm for 10 min to separate the serum. The serum was inactivated (at 56 °C for 30 min) for subsequent specific antibody detection. Detection of antibody titers against DT, TT, PT, FHA, PRN, and PRP in rat serum was performed by ELISA. Briefly, microplates were coated with antigens and blocked after washing. Test sera were diluted (pre-immunization: 100-fold; post-immunization: 100–1600-fold), subjected to serial two-fold dilutions in duplicate on the coated plates, and incubated. After washing, anti-rat IgG enzyme conjugate was added, followed by substrate incubation and reaction termination with 2M H_2_SO_4_. Optical density was measured at 450 nm. A sample was considered positive if its OD value exceeded 2.1 times that of the negative control, and seroconversion was defined as a ≥4-fold increase in the post-immunization IgG titer compared to pre-immunization levels for each antigen. Detection of neutralizing antibody titers against PV I, PV II, and PV III in rat serum was performed using a microneutralization assay. Briefly, inactivated serum samples (1:4 dilution, 56 °C for 30 min) were serially diluted two-fold across a 96-well plate. Eight replicates were performed for each sample to facilitate subsequent statistical calculations. A fixed dose of each Sabin strain virus (100 CCID_50_/50 μL) was added to the diluted serum and incubated at 35 °C for 3 h. Hep-2 cells were then added (1 × 10^5^ cells/mL) and cultured at 35 °C with 5% CO_2_. Cytopathic effect (CPE) was observed microscopically on days 2, 4, and 7. The neutralizing antibody titer was calculated using the Karber method and defined as the highest serum dilution that protected 50% of the cells from infection. A titer ≥ 1:8 was considered positive.

### 2.6. Statistical Analysis

Quantitative data (including body weight, weight gain rate [calculated as: (Body Weight − Pre-dose Weight)/Pre-dose Weight × 100%], food consumption, body temperature, hematology, coagulation, serum biochemistry, CD4^+^/CD8a^+^ T cells, cytokines, organ wet weights, and organ-to-body weight ratios) and qualitative data (urinalysis and histopathology) were tabulated using Excel. Statistical analyses were performed with TOXSTAT2006 for multi-group comparisons.

Quantitative data analysis followed a hierarchical approach: Bartlett’s test was first applied to assess variance homogeneity. If homogeneity was confirmed (*p* ≥ 0.05), ANOVA (F-test) was performed; significant ANOVA results (*p* ≤ 0.05) triggered Dunnett’s parametric test for multiple comparisons, while non-significance (*p* > 0.05) terminated the analysis. For heterogeneous variances (*p* < 0.05), the Kruskal–Wallis non-parametric test was used, with significant outcomes (*p* ≤ 0.05) followed by Dunnett’s non-parametric test, and non-significance (*p* > 0.05) concluding the analysis. This dual-path methodology ensured appropriate parametric or non-parametric inference based on initial variance characteristics.

For semi-quantitative ordinal data from urinalysis and histopathology, multi-group Ridit analysis was employed. If statistical significance was observed (*p* ≤ 0.05), further pairwise comparisons were conducted using the two-group Ridit test to identify specific inter-group differences.

## 3. Results

### 3.1. Post-Dose Clinical Signs and Local Reactions in Animals

No mortality or moribundity occurred in any groups. The negative control group showed no injection-site muscle swelling throughout the trial, while the adjuvant control group exhibited transient local swelling only on days 1–2 post fifth dose, with all cases self-resolving.

Vaccine groups displayed dose- and frequency-dependent local reactions: After the first dose, low- and high-dose groups showed muscle swelling in 32.5% (13/40) and 95% (38/40) of animals, respectively, resolving within 3 days. Post second dose, both groups had a 73% (22/30) swelling incidence, with low-dose resolving in 2 days and high-dose showing prolonged recovery. By the third dose, swelling rates were 63% (19/30) for low-dose and 40% (12/30) for high-dose, with gradual recovery during dosing intervals. After the fourth dose, swelling incidences were 37% (11/30) and 77% (23/30), with palpable induration and prolonged resolution in some rats. The fifth dose induced 60% (18/30) and 87% (26/30) swelling in low- and high-dose groups, accompanied by significantly extended recovery periods (Table 1).

### 3.2. Changes in Body Weight, Food Consumption, and Body Temperature

All groups maintained physiological stability in body weight gain throughout the study. While transient fluctuations were observed—specifically, (1) a self-limiting weight gain reduction in adjuvant control females during week 1 post second dose (Table 2, Supplement Table S1), and (2) decreased food consumption in high-dose females at week 2 post second dose (Supplement Table S2)—both events demonstrated complete reversibility within subsequent observation intervals.

Body temperature fluctuations were transient and demonstrated no clinical relevance. In male animals, significant elevations were observed in the adjuvant control, low-dose, and high-dose groups on day 1 post first dose, and in low/high-dose groups at 4–6 h post third dose. Female animals exhibited a distinct temporal pattern: (1) 4–6 h post first dose: high-dose group elevation; (2) post fourth dose: low/high-dose elevations on day 1, with only high-dose remaining elevated on day 3; (3) post fifth dose: adjuvant control and low/high-dose elevations on day 1, followed by sustained low/high-dose elevations on day 3. All other timepoints showed no statistical differences versus negative controls (Table 3). Critically, all groups demonstrated physiological temperature fluctuations within ranges consistent with the negative control group. Sporadic, mild elevations occurred in individual rats, but these exhibited no persistent or patterned trends across dosing cycles. Critically, no secondary clinical manifestations potentially linked to hyperthermia (e.g., lethargy, reduced activity, or dehydration) were observed. These data collectively confirm that transient temperature variations were self-resolving and confined within physiological tolerance, thereby excluding vaccine-related systemic effects.

### 3.3. Ophthalmic Examinations and Urinalysis

No significant abnormalities were observed in ophthalmic examinations in all groups at 2 days and 55 days after the last administration (Supplemental Table S3).

On day 2 post last dose, adjuvant control animals (both sexes) exhibited mildly reduced urine specific gravity values, though all fluctuations remained within the normal physiological range. By day 55 post last dose, adjuvant control females showed elevated specific gravity, still within normal limits; in the high-dose group, the levels of ketones, urobilinogen, and protein in the urine of male animals were elevated, which might be sporadic occurrences in individual animals (Supplement Table S4).

### 3.4. Hematology Tests

Hematological analyses revealed transient, sex-specific fluctuations: Day 3 post first dose, high-dose male rats exhibited elevated neutrophil percentage (NEU%). Day 3 post last dose, high-dose males showed an increased eosinophil count (EOS) and eosinophil percentage (EOS%), while high-dose females displayed broader alterations including elevated neutrophils (NEU), NEU%, lymphocyte percentage (LYM%), monocytes (MONO), and EOS. Concurrently, low-dose females demonstrated elevated NEU% and LYM% (Table 4).

Coagulation tests indicated elevated fibrinogen (FIB) in high-dose males/females and prolonged prothrombin time (PT) in high-dose females post first dose; post last dose, high-dose groups and low-dose females exhibited elevated FIB. These fluctuations correlated with local irritant symptoms (e.g., injection-site swelling) but were non-dose-dependent and within physiological ranges (NEU%: 40–70%, EOS: 1–6%, FIB: 1.5–3.5 g/L).

Critically, all parameters normalized to negative control levels by day 56 post-last dose, except for adjuvant control males with mildly reduced NEU% (still within normal range), confirming no persistent systemic effects (Table 4).

### 3.5. Serum Biochemistry Results: Serum Biochemical Parameters Were Analyzed on Day 3 Post First Dose, Day 3 Post Last Dose, and Day 56 Post Last Dose

All groups showed no significant differences from negative controls in liver function markers (ALT, AST, TBIL, GGT) across all timepoints. High-dose animals exhibited mildly reduced ALP vs. controls on day 3 post first dose (within physiological range), with no ALP differences at other timepoints (Table 5).

Total protein (TP) levels showed no significant differences across groups at all three timepoints. On day 3 post first dose, we observed a reduced albumin-to-globulin ratio (A/G) in adjuvant/low/high-dose groups and decreased albumin (ALB) in the high-dose group. On day 3 post last dose, we observed elevated globulin (GLO) added to persistent A/G/ALB changes (immune stimulation-linked). On day 56 post last dose, we observed full recovery, except for residual GLO and A/G deviations in high-dose females (Table 5).

Renal function (BUN, CREA), bone metabolism (calcium, phosphorus), and electrolytes (Na, Cl) remained within normal ranges without inter-group differences. Lipid/glucose metabolism showed transient TGL reduction in high-dose females post last dose (clinically insignificant; Table 5). Enzyme/electrolyte shifts included isolated CK elevation in low-dose groups post first dose and K fluctuations in low-dose/adjuvant groups post last dose (Table 5).

Critically, all fluctuations were self-limiting, non-dose-dependent, and resolved by day 56, except for minor delays in high-dose females, confirming no vaccine-related systemic toxicity (Table 5).

### 3.6. Pathological Examination

On day 3 after the last administration, 20 animals (half male and half female) were euthanized for gross organ observation, organ weighing, and histopathological examination, followed by an additional 10 animals (half male and half female) euthanized on day 56. On day 3, decreased testis-to-body weight coefficients were observed in the male adjuvant control, low-, and high-dose groups, while females in the low/high-dose groups exhibited increased thymus wet weight and body weight coefficients. By day 56, elevated spleen-to-body weight coefficients were noted in females of the adjuvant control and high-dose groups, and males in the high-dose group showed an increased brain-to-body weight coefficient (see Table 6 and Supplement Table S5).

### 3.7. CD3^+^CD4^+^ and CD3^+^CD8a^+^ T-Cell Assays and Cytokine Analysis

**Figure 2:**
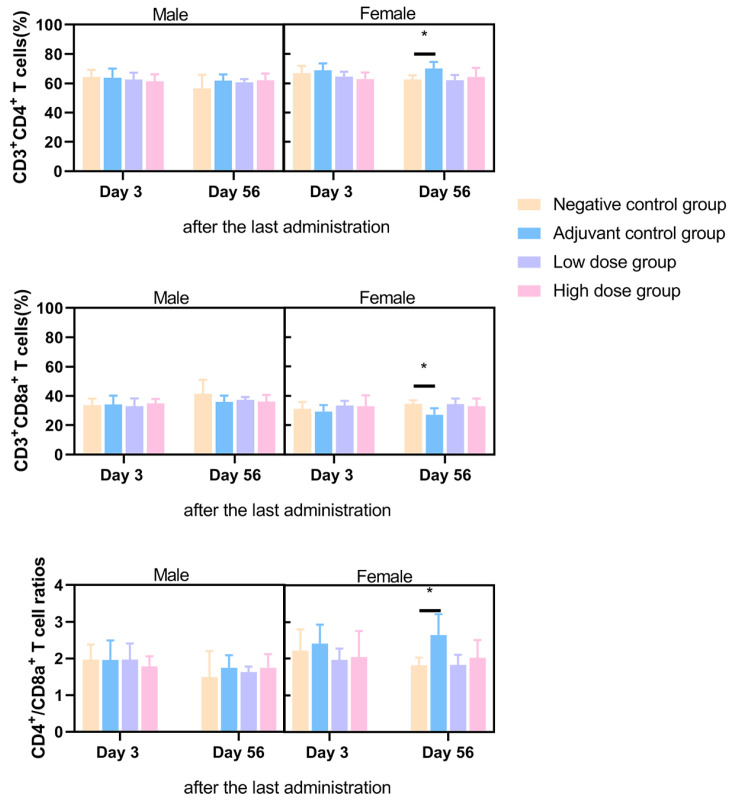
Proportions of CD3^+^CD4^+^, CD3^+^CD8a^+^, and CD4^+^CD8a^+^ T cells in peripheral blood mononuclear cells (PBMCs) of SD rats on day 3 and day 56 after the last administration.* *p* < 0.05.

Flow cytometry analysis of peripheral blood CD3^+^CD4^+^ and CD3^+^CD8a^+^ T-cell subsets was conducted at 3 days and 56 days post final administration. On day 3, no significant differences in CD4^+^/CD8^+^ ratios were detected between the adjuvant control, low-dose, or high-dose groups compared to the negative control. By day 56, females in the adjuvant control group demonstrated elevated CD4^+^ T-cell proportions, reduced CD8a^+^ cell proportions, and consequently increased CD4^+^/CD8a^+^ ratios, while vaccine groups maintained ratios within baseline ranges. These transient T-cell fluctuations in adjuvant controls were isolated to females, exhibited no dose dependency, and were attributed to spontaneous physiological variability rather than vaccine-related effects (Figure 2).

Cytokine profiles (IL-2, IFN-γ, TNF-α) at all timepoints (3 days and 56 days after the last dose) showed no differences between the vaccine group and the negative control group. Only on day 3 post the last dose were lower TNF-α levels observed in male animals of the adjuvant control group; this change was not observed in the vaccine group and had no toxicological significance (Supplement Table S6).

### 3.8. Immunogenicity

For bacterial antigens (DT, TT, PT, FHA, PRN, PRP), both low- and high-dose groups demonstrated 100% antibody seropositivity rates at 21 days post first immunization. Regarding poliovirus-specific responses, the low-dose group achieved 100% seropositivity for type I after the 2nd dose and for types II/III after the 3rd dose, whereas the high-dose group attained 100% seropositivity across all poliovirus types (I/II/III) following the 2nd dose (Supplement Table S7).

**Figure 3:**
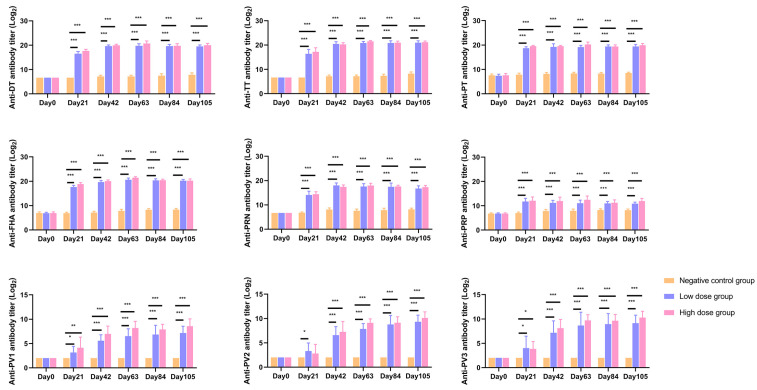
Specific antibody levels. Serum levels of specific antibodies in the satellite group of SD rats were measured before the first administration and 21 days after each administration. The vaccine components measured were as follows: Diphtheria Toxoid (DT) for the diphtheria vaccine, Tetanus Toxoid (TT) for the tetanus vaccine, Pertussis Toxin (PT), Filamentous Hemagglutinin (FHA), and Pertactin (PRN) for the pertussis vaccine, and Polyribosylribitol Phosphate (PRP) for the *Haemophilus influenzae* type b (Hib) vaccine. Antibodies against DT, TT, PT, FHA, PRN, and PRP were detected by ELISA. Antibodies against poliovirus type 1 (PV1), type 2 (PV2), and type 3 (PV3) were detected by the microneutralization assay. * *p* < 0.05; ** *p* < 0.01; *** *p* < 0.001.

Regarding antibody levels, bacterial vaccine components (DT/TT/PT/FHA/PRN/PRP) in both dose groups showed significantly higher levels (*p* < 0.01) compared to the negative control at 21 days post each immunization, with no inter-group differences. For poliovirus antibodies, after the first dose, types I and III levels were significantly elevated in both groups versus control, while type II exhibited increases in both groups but reached statistical significance only in the low-dose group (*p* < 0.05). From the 2nd to 5th doses, all poliovirus types (I/II/III) in both dose groups maintained statistically superior antibody levels compared to control (Figure 3).

## 4. Discussion

This study in SD rats demonstrated that repeated intramuscular administration of the DTacP-sIPV/Hib combined vaccine primarily induced localized and reversible muscular reactions, with no evidence of irreversible toxicity. Both low- and high-dose groups exhibited significant local swelling post initial vaccination, showing dose-dependent progression (higher incidence/severity in high-dose group) and fluctuating but controlled recurrence upon subsequent administrations—a pattern consistent with alum-adjuvanted vaccines [15], as evidenced by transient swelling in adjuvant controls after the 5th dose. Critical findings included complete resolution of all swelling within 1–3 days (maximum 21 days post-5th dose) and histopathological confirmation of ongoing tissue repair at 3/56 days post final dose, characterized by resolving multifocal chronic inflammation with residual basophilic granular deposits indicative of aluminum adjuvant clearance.

Beyond localized muscular reactions, systemic toxicity signals were minimal and predominantly adjuvant-related. Although transient body temperature elevations (notably in high-dose groups) occurred at multiple timepoints versus negative controls, fluctuations remained within physiological ranges without sustained patterns and triggered no clinical abnormalities, indicating individual stress responses rather than direct vaccine toxicity. Persistent alterations in body weight or food consumption were absent, excluding systemic vaccine effects. Hematological (e.g., NEU%, EOS, FIB) and biochemical changes (e.g., ALB, GLO, A/G) primarily in high-dose/adjuvant groups reflected alum-induced acute-phase responses, not antigen-specific toxicity. Critically, during the recovery phase at 56 days pos -final administration, all aforementioned hematological and coagulation parameters returned to levels comparable with the negative control group, confirming the transient and reversible nature of these alterations. Renal function (BUN, CREA), electrolytes (Na, Cl, K, Ca, P), and hepatic core parameters (ALT, AST, TBIL, GGT) consistently remained within normal ranges throughout the study, providing robust evidence of no adverse effects of the vaccine on vital organ functions.

Critical assessment of this combination vaccine revealed no evidence of significant immunotoxicity. Repeated administration maintained T-cell subset homeostasis, with peripheral blood CD3^+^CD4^+^/CD3^+^CD8^+^ proportions showing no significant inter-group differences at 3/56 days post final dose; the elevated CD4^+^/CD8^+^ ratio in female adjuvant controls at day 56 was attributable to spontaneous biological variation. Cytokine profiles (IL-2, IFN-γ, TNF-α) demonstrated no abnormal activation: vaccine and control groups exhibited comparable levels at both timepoints (excluding occasional below-detection values), with isolated TNF-α reduction in male adjuvant controls having no toxicological relevance, collectively indicating the absence of pathogenic inflammation. Apart from adjuvant-related local reactions at injection sites, systemic examinations of organs, including immune organs (e.g., spleen, thymus, lymph nodes), revealed no pathological alterations attributable to the adjuvant or vaccine. Observed variations such as decreased thymus wet weight/coefficient (3 days post final administration) and elevated spleen-to-body weight coefficient (56 days post final administration) demonstrated no dose dependency. These changes were classified as spontaneous or incidental variations in animals, unrelated to experimental interventions.

This vaccine, designed to provide effective multi-pathogen protection, demonstrated good immunogenicity on a foundation of favorable safety. Both low- and high-dose groups achieved rapid seroconversion, with 100% antibody seropositivity against all bacterial antigens (DT, TT, PT, FHA, PRN, PRP) by 21 days post first dose. For poliovirus responses, the high-dose group attained 100% seropositivity across all serotypes (I/II/III) after the second dose, while the low-dose group achieved 100% for type I post dose 2 and types II/III post dose 3. Antibody kinetics revealed significantly elevated levels against bacterial antigens versus negative controls from day 21 post first dose, with no statistical differences between dose groups—indicating sufficient immune induction even at the low dose. Poliovirus antibody dynamics showed early significant increases for types I/III post-first dose in both groups, and all serotypes surpassed control levels after the second dose, confirming robust immune activation. However, after the first dose, the type II poliovirus antibody level in the high-dose group showed no statistically significant difference compared to the negative control group. These results indicate that a single dose of the vaccine is insufficient to elicit high levels of poliovirus antibodies. At least two or more doses are required to achieve a high state of antibody titers against all poliovirus serotypes in rats. This phenomenon could be attributed to the intrinsic characteristics of the Sabin strain itself or to differences in immune responses to the poliovirus vaccine between SD rats and humans.

Globally established pentavalent vaccines (e.g., Pentaxim™) demonstrate the paradigm of combination vaccines in improving immunization efficiency [11]. This study focuses on the DTaP-sIPV/Hib vaccine, which possesses distinct advantages and development potential. Firstly, the sIPV produced using the Sabin strain offers potential benefits over traditional wild-strain IPV, including higher biosafety (lower production risks) and potentially lower costs. This is particularly advantageous for expanding IPV vaccination coverage globally, especially in resource-limited settings [16]. The immunogenicity data from this study strongly support that sIPV in the combined vaccine can induce highly effective protective antibodies. Secondly, this study reconfirmed that aluminum adjuvants are the primary drivers of local and partial systemic (hematological, biochemical) reactions, which aligns with current understanding of aluminum-containing vaccines [15]. Based on the above background, this vaccine employs a new aluminum adjuvant adsorption process to reduce the total aluminum adjuvant content, thereby attempting to minimize adverse reactions associated with aluminum adjuvants. Future research could explore novel adjuvants (e.g., AS series) or optimize aluminum adjuvant morphology/concentration to further improve local tolerance while maintaining strong immunogenicity. The safety record of licensed pentavalent vaccines is generally favorable, with injection site reactions (pain, redness, induration) being the primary adverse events [11,17]. The local swelling, induration, and reversible histological changes observed in this study are consistent with clinical reports, with no unexpected systemic or organ toxicity risks identified. Prolonged recovery in some animals after repeated dosing suggests close monitoring of local tolerance in clinical studies is warranted.

This study establishes a comprehensive preclinical safety package in rat models, demonstrating localized and transient reactogenicity profiles. However, species-specific responses must be considered: aluminum adjuvant-induced local reactions (e.g., swelling, granuloma) may be more pronounced in rodents than in humans [18,19]. Clinical studies should pay special attention to local reactions (pain, induration size/duration) and the incidence of fever, while also monitoring blood biochemical parameters. Particular focus should be placed on whether the changes in the albumin/globulin (A/G) ratio observed in preclinical studies are replicated in humans and their clinical implications.

In summary, the DTaP-sIPV/Hib combination vaccine demonstrated favorable safety and immunogenicity in rat models. Its safety characteristics aligned with expectations for aluminum-adjuvanted vaccines, with controllable risks, providing strong preclinical support for further clinical development. Future clinical studies should focus on human local tolerance, dose optimization (particularly exploring the feasibility of lower doses), and close monitoring of adjuvant-related hematological/biochemical parameter changes. The successful development of this vaccine is expected to offer children worldwide a safe, effective, and potentially cost-effective immunization option through combination vaccination.

## 5. Conclusions

Based on the results of the repeated-dose toxicity and immunogenicity study in SD rats, the Sabin strain-based DTaP-sIPV/Hib pentavalent vaccine demonstrated a favorable safety profile and immunogenicity. The primary adverse reaction was dose-dependent local muscle swelling, which was reversible. No irreversible damage to hepatic/renal function, coagulation, or immune organs was observed. Regarding immunogenicity, 100% seroconversion was achieved for all bacterial components 21 days after the first dose, and for poliovirus antibodies, complete seroconversion was achieved after 2–3 doses, with no significant difference in antibody levels between the low- and high-dose groups. This study confirms that the vaccine’s safety characteristics align with expectations for aluminum-adjuvanted vaccines and that it induces excellent immune responses. These findings provide critical preclinical support for subsequent clinical development, indicating that this vaccine is a promising candidate that can offer a safe, effective, and potentially cost-effective immunization option for children worldwide, particularly in resource-limited regions.

## Figures and Tables

**Table 1:** Number of rats exhibiting swelling and induration at the vaccination site.

Number of Doses	Time After Each Vaccine Dose	Negative Control Group (Occurrence/Total)	Adjuvant Control Group (Occurrence/Total)	Low-Dose Group (Occurrence/Total)	High-Dose Group (Occurrence/Total)
Before immunization		0/40	0/40	0/40	0/40
First immunization	Day 1	0/40	0/40	13/40	38/40
	Day 2	0/40	0/40	7/40	40/40
	Day 3–20	0/30	0/30	0/30	0/30
Second immunization	Day 0	0/30	0/30	0/30	0/30
	Day 1	0/30	0/30	22/30	22/30
	Day 2	0/30	0/30	0/30	3/30
	Day 3–20	0/30	0/30	0/30	2/30
Third immunization	Day 0	0/30	0/30	0/30	2/30
	Day 1	0/30	0/30	19/30	12/30
	Day 2	0/30	0/30	13/30	9/30
	Day 3	0/30	0/30	4/30	4/30
	Day 4	0/30	0/30	2/30	4/30
	Day 5–10	0/30	0/30	0/30	2/30
	Day 11–20	0/30	0/30	0/30	1/30
Fourth immunization	Day 0	0/30	0/30	0/30	1/30
	Day 1	0/30	0/30	11/30	23/30
	Day 2	0/30	0/30	10/30	20/30
	Day 3	0/30	0/30	10/30	19/30
	Day 4	0/30	0/30	4/30	18/30
	Day 5	0/30	0/30	4/30	15/30
	Day 6–7	0/30	0/30	3/30	15/30
	Day 8–11	0/30	0/30	3/30	10/30
	Day 12	0/30	0/30	1/30	9/30
	Day 13–20	0/30	0/30	0/30	3/30
Fifth immunization	Day 0	0/30	0/30	0/30	3/30
	Day 1–2	0/30	4/30	18/30	26/30
	Day 3	0/5	0/5	2/5	4/5
	Day 4–16	0/5	0/5	1/5	0/5
	Day 17–55	0/5	0/5	0/5	0/5

**Table 2:** Statistical data of weight growth rate in mice.

Number of Doses	Time After Each Vaccine Dose	Negative Control Group		Adjuvant Control Group		Low-Dose Group		High-Dose Group	
		M	F	M	F	M	F	M	F
First immunization	Week 1	20.4 ± 3.2	15.7 ± 3.9	20.5 ± 2.7	13.8 ± 5.0	20.4 ± 2.6	15.1 ± 3.2	19.7 ± 3.6	14.8 ± 3.8
	Week 2	45.2 ± 6.3	35.4 ± 7.7	42.7 ± 4.7	29.4 ± 7.8	44.5 ± 5.0	32.5 ± 7.1	43.2 ± 6.6	32.8 ± 7.6
	Week 3	66.5 ± 9.4	41.1 ± 7.6	61.4 ± 6.3	36.4 ± 6.0	64.1 ± 7.2	37.2 ± 4.7	62.3 ± 9.6	37.9 ± 6.6
Second immunization	Week 1	83.0 ± 11.3	48.8 ± 7.7	75.8 ± 8.9	42.2 ± 6.5 *	81.1 ± 9.7	49.3 ± 6.3	78.1 ± 12.1	45.4 ± 7.3
	Week 2	93.9 ± 13.3	53.9 ± 10.5	87.8 ± 10.4	47.4 ± 7.5	94.5 ± 10.5	55.8 ± 9.3	90.0 ± 13.8	49.9 ± 9.9
	Week 3	109.4 ± 14.8	60.5 ± 11.3	101.2 ± 11.5	53.7 ± 8.0	109.9 ± 12.5	59.2 ± 7.4	103.4 ± 15.0	55.5 ± 8.9
Third immunization	Week 1	117.0 ± 16.9	63.8 ± 11.1	108.3 ± 12.7	57.0 ± 8.1	116.1 ± 13.9	63.8 ± 8.4	110.4 ± 16.5	59.5 ± 9.8
	Week 2	126.2 ± 18.6	66.4 ± 13.0	116.3 ± 13.2	59.8 ± 10.8	126.8 ± 15.8	67.4 ± 10.6	118.1 ± 18.1	64.9 ± 11.2
	Week 3	132.5 ± 20.1	69.3 ± 13.2	125.0 ± 14.2	63.1 ± 10.1	135.2 ± 15.7	69.1 ± 10.2	125.4 ± 19.4	64.6 ± 10.0
Fourth immunization	Week 1	138.9 ± 20.9	76.3 ± 13.6	130.8 ± 14.2	71.2 ± 4.4	140.8 ± 17.7	73.7 ± 16.3	131.1 ± 20.0	67.1 ± 4.7
	Week 2	146.4 ± 23.3	84.9 ± 18.6	137.6 ± 15.8	74.4 ± 4.8	147.5 ± 18.5	79.9 ± 17.2	138.6 ± 21.0	70.0 ± 4.6
	Week3	152.9 ± 23.8	83.8 ± 18.3	143.3 ± 16.8	75.9 ± 5.6	155.5 ± 19.6	80.2 ± 21.0	144.7 ± 22.5	71.4 ± 6.3
Fifth immunization	Week 1	153.8 ± 23.0	84.1 ± 15.7	152.4 ± 19.5	76.2 ± 4.5	156.4 ± 11.6	79.1 ± 20.4	129.0 ± 14.2	72.3 ± 5.6
	Week 2	158.9 ± 25.1	85.2 ± 15.8	156.0 ± 20.5	77.6 ± 4.6	160.8 ± 13.3	80.8 ± 19.6	131.1 ± 13.5	72.3 ± 5.1
	Week 3	164.0 ± 26.8	87.6 ± 16.6	160.3 ± 22.2	82.0 ± 6.7	163.9 ± 15.7	84.2 ± 21.0	135.6 ± 13.6	74.0 ± 5.3
	Week 4	170.1 ± 27.5	76.3 ± 13.6	166.6 ± 19.7	71.2 ± 4.4	171.2 ± 17.6	73.7 ± 16.3	142.8 ± 14.5	67.1 ± 4.7
	Week 5	171.7 ± 26.6	90.0 ± 18.6	170.7 ± 20.6	84.4 ± 5.3	174.0 ± 17.3	89.4 ± 21.7	144.7 ± 14.8	75.1 ± 7.1
	Week 6	177.1 ± 29.0	92.4 ± 20.7	172.4 ± 20.2	82.8 ± 6.2	177.0 ± 16.9	90.0 ± 24.4	146.7 ± 16.0	77.4 ± 5.5
	Week 7	179.9 ± 28.9	93.9 ± 20.7	175.6 ± 21.8	83.6 ± 7.9	178.9 ± 18.3	90.1 ± 24.0	147.8 ± 16.7	78.0 ± 6.4
	Week 8	182.0 ± 29.7	92.6 ± 20.5	178.4 ± 20.2	86.5 ± 8.8	181.9 ± 17.5	93.2 ± 26.7	159.6 ± 9.7	79.9 ± 5.9

All groups compared with the negative control group. * *p* < 0.05. M: Male; F: Female. The numbers in the table represent the mean ± SD of the weight growth rate. Weight Growth Rate = (Current Weight—Pre-treatment Weight)/Pre-treatment Weight × 100%.

**Table 3:** Statistical data of animal body temperature (℃).

Number of Doses	Time After Each Vaccine Dose	Negative Control Group		Adjuvant Control Group		Low-Dose Group		High-Dose Group	
		M	F	M	F	M	F	M	F
First immunization	4–6 h	37.1 ± 0.5	37.2 ± 0.3	37.2 ± 0.3	37.3 ± 0.4	37.1 ± 0.5	37.4 ± 0.5	36.9 ± 0.3	37.6 ± 0.4 **
	Day 1	37.1 ± 0.6	37.7 ± 0.5	36.7 ± 0.4 **	37.9 ± 0.7	37.4 ± 0.4 **	38.2 ± 0.6	37.4 ± 0.4 **	38.1 ± 0.5
	Day 3	37.6 ± 0.6	37.6 ± 0.4	37.4 ± 0.4	38.1 ± 0.5	37.3 ± 0.4	37.8 ± 0.7	37.2 ± 0.3	37.9 ± 0.5
Second immunization	4–6 h	36.7 ± 0.3	36.9 ± 0.3	36.7 ± 0.4	36.9 ± 0.3	36.9 ± 0.6	36.8 ± 0.3	37.0 ± 0.5	37.2 ± 0.3
	Day 1	37.1 ± 0.5	38.0 ± 0.5	36.9 ± 0.4	37.8 ± 0.7	37.3 ± 0.5	38.3 ± 0.5	37.1 ± 0.4	38.2 ± 0.8
Third immunization	4–6 h	36.4 ± 0.4	37.4 ± 0.5	36.7 ± 0.4	37.2 ± 0.7	37.0 ± 0.5 **	37.0 ± 0.5	36.9 ± 0.3 **	37.8 ± 0.7
	Day 1	36.9 ± 0.6	38.2 ± 0.4	37.3 ± 0.7	38.0 ± 0.5	37.0 ± 0.7	37.8 ± 0.6	37.1 ± 0.7	38.3 ± 0.7
Fourth immunization	4–6 h	36.7 ± 0.3	37.0 ± 0.4	36.8 ± 0.3	37.5 ± 0.7	36.7 ± 0.3	37.2 ± 0.6	36.8 ± 0.3	37.4 ± 0.5
	Day 1	36.9 ± 0.5	38.0 ± 0.3	37.2 ± 0.7	38.2 ± 0.5	37.0 ± 0.6	38.6 ± 0.6 **	36.8 ± 0.5	38.5 ± 0.6 *
	Day 3		38.1 ± 0.3		37.8 ± 0.6		38.0 ± 0.6		37.4 ± 0.7 *
Fifth immunization	4–6 h	36.5 ± 0.4	37.3 ± 0.4	36.5 ± 0.4	37.5 ± 0.9	36.4 ± 0.6	37.6 ± 0.8	36.7 ± 0.4	38.0 ± 0.7
	Day 1	36.4 ± 0.5	37.0 ± 0.6	36.5 ± 0.8	38.1 ± 0.6 **	36.6 ± 0.7	38.2 ± 0.6 **	35.9 ± 0.6	38.2 ± 0.7 **
	Day 3		37.3 ± 0.7		38.1 ± 0.6		38.6 ± 0.3 **		38.4 ± 0.5 *

All groups compared with the negative control group. * *p* < 0.05; ** *p* < 0.01. M: Male; F: Female. The numbers in the table represent the mean ± SD.

**Table 4:** Statistical data of hematology and coagulation tests.

Time After Each Vaccine Dose			Negative Control Group		Adjuvant Control Group		Low-Dose Group		High-Dose Group	
			M	F	M	F	M	F	M	F
3 days after the first administration	WBC	(×10^9^/L)	6.85 ± 1.80	6.15 ± 1.14	6.09 ± 1.50	5.86 ± 0.93	6.48 ± 1.84	6.21 ± 0.79	6.65 ± 0.80	5.87 ± 0.86
	NEU	(×10^9^/L)	0.88 ± 0.30	0.70 ± 0.14	0.76 ± 0.22	0.81 ± 0.33	0.72 ± 0.15	0.61 ± 0.16	1.10 ± 0.10	0.73 ± 0.06
	NEU%	(%)	12.9 ± 3.0	11.4 ± 0.4	12.3 ± 1.7	13.9 ± 5.1	11.5 ± 1.7	10.1 ± 3.3	16.6 ± 2.1 *	12.7 ± 2.8
	LYM	(×10^9^/L)	5.39 ± 1.54	4.99 ± 0.93	4.81 ± 1.22	4.61 ± 0.90	5.27 ± 1.61	5.17 ± 0.92	4.87 ± 0.62	4.66 ± 0.84
	LYM%	(%)	78.4 ± 4.4	81.0 ± 1.2	79.1 ± 3.3	78.5 ± 7.1	80.9 ± 2.4	82.8 ± 4.0	73.3 ± 2.1	79.1 ± 3.1
	MONO	(×10^9^/L)	0.53 ± 0.15	0.38 ± 0.07	0.46 ± 0.14	0.37 ± 0.13	0.43 ± 0.09	0.35 ± 0.04	0.60 ± 0.15	0.39 ± 0.07
	MONO%	(%)	7.9 ± 2.3	6.3 ± 1.1	7.6 ± 1.5	6.3 ± 1.9	6.8 ± 1.0	5.7 ± 0.8	8.9 ± 1.3	6.7 ± 0.9
	EOS	(×10^9^/L)	0.04 ± 0.01	0.07 ± 0.03	0.05 ± 0.02	0.07 ± 0.01	0.04 ± 0.01	0.07 ± 0.02	0.06 ± 0.01	0.08 ± 0.03
	EOS%	(%)	0.7 ± 0.3	1.1 ± 0.3	0.8 ± 0.3	1.1 ± 0.2	0.7 ± 0.2	1.2 ± 0.2	1.0 ± 0.2	1.3 ± 0.3
	BASO	(×10^9^/L)	0.01 ± 0.00	0.01 ± 0.00	0.01 ± 0.00	0.01 ± 0.01	0.01 ± 0.01	0.01 ± 0.00	0.01 ± 0.01	0.01 ± 0.00
	BASO%	(%)	0.2 ± 0.1	0.2 ± 0.1	0.1 ± 0.1	0.1 ± 0.1	0.1 ± 0.1	0.1 ± 0.1	0.2 ± 0.1	0.2 ± 0
	RBC	(×10^12^/L)	6.24 ± 0.38	6.51 ± 0.20	6.12 ± 0.22	6.58 ± 0.41	6.08 ± 0.24	6.49 ± 0.23	6.26 ± 0.43	6.27 ± 0.35
	HGB	(g/dL)	13.2 ± 0.8	13.6 ± 0.3	13.0 ± 0.6	13.7 ± 0.8	12.9 ± 0.6	13.5 ± 0.9	13.0 ± 0.7	13.2 ± 0.7
	HCT	(%)	39.4 ± 2.0	39.7 ± 0.9	38.9 ± 1.5	39.6 ± 2.0	38.6 ± 1.6	39.2 ± 2.0	38.9 ± 1.7	38.4 ± 2.1
	MCV	(fL)	63.2 ± 1.3	61.0 ± 0.7	63.5 ± 1.4	60.2 ± 1.1	63.5 ± 2.5	60.4 ± 1.1	62.3 ± 1.7	61.2 ± 1.3
	MCH	(pg)	21.1 ± 0.6	20.9 ± 0.5	21.2 ± 0.6	20.9 ± 0.5	21.2 ± 1.1	20.8 ± 0.7	20.8 ± 0.4	21.1 ± 0.4
	MCHC	(g/dL)	33.4 ± 0.6	34.3 ± 0.5	33.4 ± 0.5	34.6 ± 0.3	33.4 ± 0.5	34.4 ± 0.7	33.4 ± 0.4	34.5 ± 0.2
	RDW	(%)	12.3 ± 1.0	10.6 ± 0.3	12.0 ± 0.5	10.8 ± 0.2	12.2 ± 0.3	10.6 ± 0.3	11.8 ± 0.6	10.4 ± 0.2
	PLT	(×10^9^/L)	1278 ± 68	1262 ± 74	1280 ± 60	1173 ± 197	1317 ± 87	1290 ± 91	1442 ± 153	1259 ± 148
	MPV	(fL)	7.3 ± 0.2	7.3 ± 0.3	7.1 ± 0.2	7.3 ± 0.2	7.3 ± 0.1	7.2 ± 0.2	7.3 ± 0.2	7.1 ± 0.1
	RETIC%	(%)	8.59 ± 1.19	5.12 ± 0.62	7.48 ± 0.83	5.01 ± 0.93	7.99 ± 0.24	4.76 ± 0.38	7.75 ± 1.13	4.56 ± 0.96
	RETIC	(×10^9^/L)	532.9 ± 46.4	333.6 ± 44.2	458.8 ± 64.6	327.6 ± 47.4	485.5 ± 23.9	309.6 ± 30.4	483.3 ± 64.3	284.9 ± 55.2
	PT	(S)	13.1 ± 0.7	12.5 ± 0.7	12.6 ± 0.5	13.0 ± 0.4	13.0 ± 0.6	12.7 ± 0.5	13.6 ± 1.0	13.9 ± 0.8 *
	APTT	(S)	13.3 ± 1.7	11.6 ± 1.8	13.2 ± 1.1	12.4 ± 1.4	13.0 ± 0.7	13.3 ± 1.1	13.7 ± 1.6	13.1 ± 1.2
	FIB	(mg/dL)	188 ± 23	170 ± 6	184 ± 10	171 ± 7	184 ± 5	176 ± 15	218 ± 20 *	196 ± 4 **
3 days after the last administration	WBC	(×10^9^/L)	9.14 ± 3.15	5.06 ± 1.56	7.98 ± 2.29	4.74 ± 1.29	7.39 ± 2.93	5.28 ± 0.96	9.19 ± 2.70	6.42 ± 2.23
	NEU	(×10^9^/L)	1.32 ± 0.35	0.73 ± 0.31	1.88 ± 1.26	0.89 ± 0.46	1.51 ± 0.31	1.15 ± 0.36	1.87 ± 0.81	1.33 ± 0.43 **
	NEU%	(%)	15.8 ± 5.7	14.2 ± 4.3	22.8 ± 9.5	18.8 ± 7.9	23.0 ± 10.1	21.8 ± 5.4 *	21.0 ± 7.2	21.9 ± 7.0 *
	LYM	(×10^9^/L)	7.17 ± 2.83	3.94 ± 1.25	5.40 ± 1.53	3.46 ± 1.05	5.16 ± 2.37	3.64 ± 0.79	6.45 ± 2.24	4.44 ± 1.89
	LYM%	(%)	76.8 ± 7.1	77.9 ± 4.8	68.6 ± 10.3	73.0 ± 8.3	67.6 ± 8.7	69.1 ± 7.2 *	69.8 ± 8.3	67.7 ± 7.5 **
	MONO	(×10^9^/L)	0.53 ± 0.17	0.29 ± 0.10	0.57 ± 0.26	0.30 ± 0.11	0.59 ± 0.32	0.36 ± 0.12	0.68 ± 0.30	0.47 ± 0.16 **
	MONO%	(%)	6.0 ± 1.5	5.9 ± 1.4	7.0 ± 2.2	6.3 ± 1.2	7.6 ± 1.9	6.8 ± 1.4	7.2 ± 1.6	7.4 ± 1.5
	EOS	(×10^9^/L)	0.11 ± 0.04	0.09 ± 0.03	0.11 ± 0.04	0.08 ± 0.02	0.12 ± 0.05	0.12 ± 0.07	0.17 ± 0.05 *	0.17 ± 0.06 **
	EOS%	(%)	1.3 ± 0.5	1.8 ± 0.6	1.5 ± 0.4	1.8 ± 0.7	1.7 ± 0.6	2.2 ± 1.1	1.9 ± 0.3 *	2.8 ± 1.2
	BASO	(×10^9^/L)	0.01 ± 0.01	0.01 ± 0	0.01 ± 0.01	0.01 ± 0.01	0.01 ± 0.01	0.01 ± 0	0.02 ± 0.01	0.01 ± 0
	BASO%	(%)	0.2 ± 0.1	0.2 ± 0.1	0.2 ± 0.1	0.1 ± 0.1	0.2 ± 0.1	0.2 ± 0.1	0.2 ± 0.1	0.2 ± 0.1
	RBC	(×10^12^/L)	8.83 ± 0.34	7.90 ± 0.41	8.74 ± 0.59	7.72 ± 0.33	8.55 ± 0.76	7.91 ± 0.38	8.79 ± 0.39	8.02 ± 0.33
	HGB	(g/dL)	15.6 ± 0.7	14.7 ± 0.4	15.3 ± 0.7	14.5 ± 0.6	14.9 ± 1.4	14.7 ± 0.7	15.4 ± 0.5	14.7 ± 0.8
	HCT	(%)	46.4 ± 2.1	42.6 ± 1.3	45.3 ± 1.9	41.9 ± 1.5	44.5 ± 4.3	42.7 ± 2.2	45.8 ± 1.6	42.7 ± 2.4
	MCV	(fL)	52.6 ± 1.3	54.0 ± 2.3	52.0 ± 2.1	54.3 ± 2.0	52.0 ± 1.6	54.0 ± 1.5	52.1 ± 1.3	53.3 ± 1.7
	MCH	(pg)	17.7 ± 0.5	18.7 ± 1.0	17.5 ± 0.8	18.9 ± 0.8	17.4 ± 0.5	18.6 ± 0.6	17.5 ± 0.5	18.3 ± 0.6
	MCHC	(g/dL)	33.6 ± 0.5	34.6 ± 0.4	33.6 ± 0.4	34.7 ± 0.4	33.5 ± 0.4	34.4 ± 0.4	33.6 ± 0.4	34.3 ± 0.3
	RDW	(%)	15.7 ± 0.9	12.9 ± 0.9	15.6 ± 1.2	12.4 ± 1.1	15.3 ± 1.2	12.9 ± 1.2	15.8 ± 1.2	13.1 ± 0.7
	PLT	(×10^9^/L)	1245 ± 185	1165 ± 81	1205 ± 109	1209 ± 175	1236 ± 297	1207 ± 131	1285 ± 127	1274 ± 78
	MPV	(fL)	7.5 ± 0.2	7.4 ± 0.3	7.5 ± 0.3	7.4 ± 0.3	7.4 ± 0.4	7.5 ± 0.3	7.3 ± 0.2	7.2 ± 0.2
	RETIC%	(%)	2.85 ± 0.60	2.66 ± 0.23	2.79 ± 0.30	2.45 ± 0.42	2.76 ± 0.44	2.70 ± 0.50	2.86 ± 0.32	2.87 ± 0.56
	RETIC	(×10^9^/L)	251.1 ± 51.2	209.9 ± 19.1	243.0 ± 23.3	189.7 ± 37.2	235.0 ± 39.7	213.8 ± 41.8	251.3 ± 29.2	229.0 ± 39.5
	PT	(S)	13.1 ± 1.0	11.3 ± 0.8	13.3 ± 1.3	11.0 ± 0.5	13.7 ± 1.8	11.2 ± 0.8	13.8 ± 1.1	11.7 ± 0.6
	APTT	(S)	15.8 ± 1.0	14.7 ± 0.9	16.0 ± 1.2	14.3 ± 0.7	15.9 ± 1.5	14.9 ± 1.2	15.3 ± 1.2	15.0 ± 1.4
	FIB	(mg/dL)	191 ± 14	149 ± 9	191 ± 11	163 ± 14	202 ± 24	183 ± 16 **	213 ± 14 *	199 ± 16 **
56 days after the last administration	WBC	(×10^9^/L)	6.77 ± 1.99	3.09 ± 0.92	8.18 ± 2.16	3.61 ± 1.22	5.65 ± 1.16	3.60 ± 1.37	6.51 ± 3.68	3.48 ± 1.40
	NEU	(×10^9^/L)	1.81 ± 0.86	0.50 ± 0.10	1.23 ± 0.30	0.62 ± 0.31	1.37 ± 0.32	0.72 ± 0.26	1.96 ± 1.21	0.69 ± 0.28
	NEU%	(%)	26.3 ± 7.7	17.2 ± 5.4	15.5 ± 3.5 *	17.3 ± 5.7	24.5 ± 4.8	20.8 ± 4.4	29.8 ± 8.6	20.5 ± 6.9
	LYM	(×10^9^/L)	4.38 ± 1.22	2.30 ± 0.89	6.36 ± 1.92	2.68 ± 1.01	3.76 ± 0.88	2.56 ± 1.11	4.04 ± 2.50	2.43 ± 1.14
	LYM%	(%)	65.1 ± 8.6	73.0 ± 8.0	77.0 ± 4.9	73.9 ± 6.2	66.3 ± 4.9	69.9 ± 6.4	61.8 ± 12.0	68.9 ± 6.0
	MONO	(×10^9^/L)	0.46 ± 0.12	0.22 ± 0.05	0.44 ± 0.09	0.22 ± 0.05	0.41 ± 0.11	0.24 ± 0.06	0.40 ± 0.20	0.25 ± 0.06
	MONO%	(%)	7.0 ± 1.2	7.5 ± 2.1	5.6 ± 1.5	6.4 ± 1.1	7.4 ± 1.4	7.0 ± 1.6	6.5 ± 2.9	7.4 ± 1.3
	EOS	(×10^9^/L)	0.11 ± 0.05	0.06 ± 0.01	0.14 ± 0.05	0.09 ± 0.04	0.10 ± 0.01	0.08 ± 0.03	0.10 ± 0.03	0.10 ± 0.06
	EOS%	(%)	1.6 ± 0.5	2.2 ± 1.1	1.8 ± 0.9	2.3 ± 0.6	1.8 ± 0.4	2.2 ± 0.7	1.7 ± 0.7	2.9 ± 0.6
	BASO	(×10^9^/L)	0.01 ± 0.01	0.0 ± 0.0	0.01 ± 0.01	0.0 ± 0.01	0.01 ± 0.01	0.0 ± 0.0	0.01 ± 0.01	0.01 ± 0.0
	BASO%	(%)	0.1 ± 0.1	0.1 ± 0.2	0.2 ± 0.1	0.1 ± 0.1	0.1 ± 0.1	0 ± 0.1	0.2 ± 0.1	0.3 ± 0.2
	RBC	(×10^12^/L)	8.60 ± 0.45	7.68 ± 0.13	8.86 ± 0.56	7.54 ± 0.30	9.06 ± 0.58	7.47 ± 0.16	8.79 ± 0.55	7.67 ± 0.37
	HGB	(g/dL)	15.0 ± 1.0	14.2 ± 0.2	15.0 ± 0.4	13.9 ± 0.4	15.3 ± 0.7	13.8 ± 0.3	14.9 ± 0.5	13.9 ± 0.6
	HCT	(%)	44.6 ± 2.3	41.2 ± 0.8	44.7 ± 1.2	40.4 ± 1.1	46.0 ± 2.2	40.9 ± 0.8	45.0 ± 1.7	41.0 ± 1.8
	MCV	(fL)	51.8 ± 0.2	53.7 ± 1.6	50.5 ± 2.3	53.6 ± 1.0	50.8 ± 1.6	54.7 ± 1.0	51.2 ± 1.3	53.4 ± 0.7
	MCH	(pg)	17.5 ± 0.3	18.5 ± 0.5	17.0 ± 0.9	18.5 ± 0.3	16.9 ± 0.7	18.5 ± 0.3	17.0 ± 0.5	18.2 ± 0.3
	MCHC	(g/dL)	33.7 ± 0.6	34.4 ± 0.3	33.6 ± 0.3	34.4 ± 0.4	33.3 ± 0.4	33.8 ± 0.6	33.1 ± 0.2	34.0 ± 0.2
	RDW	(%)	16.2 ± 0.5	12.2 ± 0.9	16.5 ± 0.9	12.8 ± 0.5	16.6 ± 1.0	12.2 ± 0.7	16.5 ± 0.6	12.8 ± 0.8
	PLT	(×10^9^/L)	1377 ± 176	1034 ± 147	1253 ± 98	1051 ± 123	1361 ± 193	999 ± 98	1167 ± 144	1072 ± 131
	MPV	(fL)	7.1 ± 0.2	7.5 ± 0.3	7.2 ± 0.1	7.5 ± 0.3	7.0 ± 0.2	7.9 ± 0.2	7.2 ± 0.2	7.7 ± 0.1
	RETIC%	(%)	3.09 ± 0.33	2.47 ± 0.43	3.01 ± 0.46	2.63 ± 0.34	2.98 ± 0.53	2.07 ± 0.43	2.81 ± 0.31	2.49 ± 0.17
	RETIC	(×10^9^/L)	265.0 ± 26.3	189.4 ± 31.6	266.9 ± 45.5	197.6 ± 21.5	269.4 ± 43.5	154.7 ± 31.2	246.3 ± 22.0	190.9 ± 9.9
	PT	(S)	14.1 ± 3.2	14.3 ± 6.8	12.2 ± 0.6	11.4 ± 1.0	13.2 ± 2.6	11.0 ± 1.4	14.1 ± 0.6	11.2 ± 0.5
	APTT	(S)	16.5 ± 1.9	14.5 ± 0.6	16.8 ± 1.8	16.9 ± 3.3	16.5 ± 2.1	15.3 ± 1.9	19.3 ± 5.8	16.8 ± 2.4
	FIB	(mg/dL)	180 ± 19	131 ± 8	166 ± 8	131 ± 6	171 ± 9	131 ± 10	158 ± 8	134 ± 14

M: Male; F: Female. All groups compared with the negative control group. * *p* < 0.05; ** *p* < 0.01. The numbers in the table represent the mean ± SD.

**Table 5:** Statistical data of serum biochemical tests.

Time After Each Vaccine Dose			Negative Control Group		Adjuvant Control Group		Low-Dose Group		High-Dose Group	
			M	F	M	F	M	F	M	F
3 days after the first administration	AST	(U/L)	84 ± 7	87 ± 10	86 ± 6	89 ± 10	77 ± 3	90 ± 11	80 ± 7	85 ± 11
	ALT	(U/L)	45 ± 5	28 ± 8	42 ± 3	29 ± 7	42 ± 6	27 ± 7	42 ± 4	27 ± 4
	ALP	(U/L)	357 ± 65	184 ± 45	322 ± 61	142 ± 34	277 ± 52	154 ± 28	254 ± 33 *	120 ± 6 **
	TBIL	(μmol/L)	3.39 ± 1.21	2.85 ± 0.49	3.95 ± 0.46	2.73 ± 0.67	3.42 ± 0.17	3.36 ± 0.97	3.40 ± 0.79	2.83 ± 0.51
	TP	(g/L)	58.5 ± 1.8	59.1 ± 2.6	57.6 ± 3.2	58.5 ± 1.9	56.4 ± 2.7	57.7 ± 3.5	57.3 ± 2.2	56.2 ± 2.2
	ALB	(g/L)	29.2 ± 0.7	30.0 ± 1.4	27.9 ± 1.6	28.6 ± 1.0	26.9 ± 2.0	27.9 ± 1.9	26.3 ± 1.0 *	25.9 ± 1.2 **
	GLO	(g/L)	29.3 ± 1.6	29.1 ± 1.7	29.8 ± 1.6	29.9 ± 1.0	29.4 ± 1.3	29.7 ± 1.7	30.9 ± 1.2	30.3 ± 1.2
	A/G		1.00 ± 0.06	1.03 ± 0.06	0.94 ± 0.01	0.96 ± 0.02 *	0.92 ± 0.07 *	0.94 ± 0.03 **	0.85 ± 0.02 **	0.85 ± 0.03 **
	BUN	(mmol/L)	4.9 ± 0.8	4.5 ± 0.8	3.6 ± 1.0	4.1 ± 0.8	5.0 ± 1.3	4.3 ± 0.9	4.3 ± 0.8	4.7 ± 1.1
	CREA	(μmol/L)	34 ± 3	39 ± 4	37 ± 2	37 ± 5	34 ± 5	39 ± 8	34 ± 1	39 ± 3
	CHOL	(mmol/L)	2.36 ± 0.26	1.88 ± 0.41	2.47 ± 0.26	2.05 ± 0.34	2.77 ± 0.43	2.07 ± 0.30	2.21 ± 0.12	1.81 ± 0.27
	TGL	(mmol/L)	0.82 ± 0.17	0.50 ± 0.08	0.78 ± 0.15	0.50 ± 0.09	0.88 ± 0.25	0.59 ± 0.19	0.88 ± 0.28	0.48 ± 0.07
	GLU	(mmol/L)	5.41 ± 0.71	5.84 ± 0.23	5.64 ± 0.62	6.08 ± 0.23	5.75 ± 0.57	6.25 ± 0.44	5.56 ± 0.61	6.06 ± 0.40
	CK	(U/L)	259 ± 40	186 ± 21	282 ± 38	197 ± 14	303 ± 69	251 ± 32 **	272 ± 53	203 ± 21
	K	(mmol/L)	4.4 ± 0.2	4.4 ± 0.4	4.4 ± 0.1	4.4 ± 0.2	4.7 ± 0.1	4.2 ± 0.2	4.6 ± 0.3	4.5 ± 0.2
	NA	(mmol/L)	143 ± 2	141 ± 9	144 ± 1	146 ± 1	143 ± 1	137 ± 7	144 ± 1	142 ± 4
	CL	(mmol/L)	104 ± 1	105 ± 2	103 ± 1	104 ± 1	103 ± 1	106 ± 2	104 ± 2	105 ± 1
	GGT	(U/L)	0.5 ± 0.9	0.1 ± 0.2	0.5 ± 0.8	0.0 ± 0.0	0.1 ± 0.2	0.3 ± 0.3	0.3 ± 0.4	0.4 ± 0.5
	CA	(mmol/L)	2.80 ± 0.11	2.73 ± 0.03	2.73 ± 0.10	2.72 ± 0.02	2.77 ± 0.06	2.63 ± 0.11	2.77 ± 0.09	2.66 ± 0.08
	PHOS	(mmol/L)	3.35 ± 0.14	3.13 ± 0.20	3.32 ± 0.15	2.94 ± 0.22	3.41 ± 0.19	3.13 ± 0.40	3.44 ± 0.16	3.05 ± 0.22
3 days after the last administration	AST	(U/L)	100 ± 15	85 ± 8	96 ± 13	95 ± 18	115 ± 31	109 ± 52	126 ± 66	95 ± 13
	ALT	(U/L)	39 ± 8	29 ± 5	42 ± 13	34 ± 9	38 ± 5	44 ± 24	51 ± 31	30 ± 6
	ALP	(U/L)	77 ± 11	30 ± 8	89 ± 17	36 ± 6	77 ± 11	37 ± 8	78 ± 16	37 ± 9
	TBIL	(μmol/L)	2.61 ± 0.75	3.35 ± 1.04	2.30 ± 0.69	2.95 ± 1.09	2.49 ± 1.21	3.20 ± 1.15	2.46 ± 1.35	2.85 ± 0.96
	TP	(g/L)	65.7 ± 2.3	72.9 ± 4.6	66.0 ± 3.0	72.0 ± 4.0	68.0 ± 4.4	73.7 ± 5.0	67.9 ± 2.3	69.9 ± 4.7
	ALB	(g/L)	31.6 ± 1.5	37.9 ± 2.6	31.4 ± 1.1	37.4 ± 3.0	30.9 ± 1.9	36.1 ± 3.6	29.8 ± 1.0 *	32.0 ± 2.9 **
	GLO	(g/L)	34.2 ± 1.5	35.0 ± 2.7	34.7 ± 2.3	34.6 ± 1.9	37.1 ± 3.2 *	37.7 ± 1.9 *	38.1 ± 2.6 **	37.9 ± 2.3 *
	A/G		0.93 ± 0.06	1.09 ± 0.08	0.91 ± 0.05	1.09 ± 0.09	0.84 ± 0.08 *	0.96 ± 0.07 **	0.79 ± 0.07 **	0.84 ± 0.07 **
	BUN	(mmol/L)	4.2 ± 0.7	5.4 ± 0.3	4.5 ± 0.9	5.6 ± 0.5	4.7 ± 1.0	5.7 ± 1.0	4.4 ± 1.0	6.2 ± 1.3
	CREA	(μmol/L)	69 ± 20	56 ± 8	62 ± 15	53 ± 6	65 ± 13	62 ± 13	61 ± 18	69 ± 20
	CHOL	(mmol/L)	2.11 ± 0.47	2.46 ± 0.34	2.29 ± 0.42	2.57 ± 0.39	2.36 ± 0.62	2.48 ± 0.44	2.15 ± 0.35	2.11 ± 0.51
	TGL	(mmol/L)	0.86 ± 0.45	0.90 ± 0.47	0.90 ± 0.40	0.66 ± 0.24	1.00 ± 0.34	0.72 ± 0.24	0.74 ± 0.25	0.53 ± 0.17 **
	GLU	(mmol/L)	7.73 ± 0.78	7.06 ± 0.76	8.14 ± 0.58	6.99 ± 0.55	7.71 ± 0.98	7.79 ± 0.83	7.89 ± 1.22	7.34 ± 1.14
	CK	(U/L)	308 ± 118	151 ± 35	245 ± 94	171 ± 64	619 ± 878	187 ± 82	300 ± 129	220 ± 174
	K	(mmol/L)	4.3 ± 0.3	4.2 ± 0.4	4.4 ± 0.3	4.1 ± 0.3	4.8 ± 0.6 *	4.0 ± 0.4	4.3 ± 0.3	4.0 ± 0.4
	NA	(mmol/L)	143 ± 3	142 ± 1	144 ± 2	143 ± 2	144 ± 2	142 ± 2	143 ± 2	143 ± 1
	CL	(mmol/L)	103 ± 1	103 ± 2	104 ± 1	104 ± 2	103 ± 1	103 ± 2	102 ± 1	104 ± 2
	GGT	(U/L)	0.3 ± 0.6	0 ± 0	0 ± 0	0.1 ± 0.2	0 ± 0	0.1 ± 0.2	0.4 ± 0.9	0.8 ± 2.0
	CA	(mmol/L)	2.60 ± 0.13	2.69 ± 0.14	2.65 ± 0.12	2.71 ± 0.08	2.52 ± 0.20	2.70 ± 0.16	2.59 ± 0.07	2.63 ± 0.10
	PHOS	(mmol/L)	2.35 ± 0.32	1.90 ± 0.17	2.19 ± 0.16	1.89 ± 0.22	2.30 ± 0.34	1.93 ± 0.24	2.21 ± 0.26	2.10 ± 0.40
56 days after the last administration	AST	(U/L)	84 ± 14	59 ± 8	95 ± 14	92 ± 49	80 ± 18	74 ± 18	94 ± 30	83 ± 25
	ALT	(U/L)	42 ± 9	27 ± 5	44 ± 2	43 ± 32	39 ± 8	35 ± 8	42 ± 12	39 ± 17
	ALP	(U/L)	70 ± 21	36 ± 9	88 ± 3	34 ± 7	63 ± 9	31 ± 16	89 ± 26	29 ± 7
	TBIL	(μmol/L)	3.30 ± 0.78	3.82 ± 2.12	4.14 ± 0.60	3.77 ± 1.22	3.73 ± 1.16	3.39 ± 1.08	3.36 ± 1.13	3.20 ± 1.04
	TP	(g/L)	67.5 ± 4.0	73.9 ± 4.3	65.7 ± 4.4	73.6 ± 7.5	69.8 ± 4.4	78.4 ± 5.0	66.6 ± 0.9	81.1 ± 6.7
	ALB	(g/L)	32.0 ± 2.0	40.6 ± 3.1	32.3 ± 1.8	40.0 ± 4.4	32.0 ± 2.2	41.4 ± 4.2	31.2 ± 0.8	39.2 ± 1.9
	GLO	(g/L)	35.5 ± 2.4	33.3 ± 1.5	33.4 ± 2.7	33.7 ± 3.6	37.8 ± 2.9	37.0 ± 2.3	35.4 ± 1.4	42.0 ± 6.6 **
	A/G		0.90 ± 0.05	1.22 ± 0.07	0.97 ± 0.03	1.19 ± 0.09	0.85 ± 0.06	1.12 ± 0.13	0.89 ± 0.05	0.95 ± 0.16 **
	BUN	(mmol/L)	4.2 ± 0.8	5.6 ± 1.5	5.2 ± 0.4	6.5 ± 0.9	4.7 ± 1.1	5.0 ± 1.0	4.9 ± 0.4	6.9 ± 0.8
	CREA	(μmol/L)	55 ± 5	54 ± 6	55 ± 12	53 ± 3	53 ± 15	56 ± 3	53 ± 17	58 ± 10
	CHOL	(mmol/L)	2.43 ± 0.51	2.76 ± 0.40	2.51 ± 0.54	2.57 ± 0.82	2.49 ± 0.36	2.89 ± 0.63	2.14 ± 0.18	2.98 ± 0.70
	TGL	(mmol/L)	0.90 ± 0.37	0.74 ± 0.24	0.77 ± 0.37	0.87 ± 0.64	0.88 ± 0.41	0.62 ± 0.38	0.40 ± 0.15	0.42 ± 0.19
	GLU	(mmol/L)	6.68 ± 0.62	6.80 ± 0.84	7.06 ± 1.15	6.79 ± 0.42	6.69 ± 1.14	6.94 ± 0.62	7.27 ± 0.85	6.82 ± 0.30
	CK	(U/L)	252 ± 131	103 ± 29	518 ± 184	104 ± 44	366 ± 179	99 ± 30	295 ± 139	127 ± 53
	K	(mmol/L)	4.6 ± 0.4	3.9 ± 0.1	5.1 ± 0.4 **	3.9 ± 0.5	4.9 ± 0.3	4.0 ± 0.2	4.5 ± 0	4.2 ± 0.3
	NA	(mmol/L)	142 ± 2	141 ± 1	143 ± 2	143 ± 2	143 ± 1	142 ± 2	145 ± 2	142 ± 2
	CL	(mmol/L)	103 ± 3	104 ± 1	104 ± 1	104 ± 2	103 ± 1	103 ± 2	105 ± 3	104 ± 2
	GGT	(U/L)	4.1 ± 0.7	5.7 ± 1.6	4.3 ± 0.5	5.1 ± 0.8	4.8 ± 1.0	6.1 ± 0.4	3.9 ± 2.4	5.5 ± 2.2
	CA	(mmol/L)	2.71 ± 0.18	2.82 ± 0.10	2.69 ± 0.09	2.79 ± 0.13	2.69 ± 0.06	2.83 ± 0.13	2.61 ± 0.04	2.82 ± 0.16
	PHOS	(mmol/L)	1.96 ± 0.13	1.76 ± 0.15	2.15 ± 0.18	1.75 ± 0.26	2.07 ± 0.15	1.76 ± 0.43	2.09 ± 0.25	1.82 ± 0.15

M: Male; F: Female. All groups compared with the negative control group. * *p* < 0.05; ** *p* < 0.01. The numbers in the table represent the mean ± SD.

**Table 6:** Statistical Data of Organ-to-Body Weight Coefficients in Animals.

Time After Each Vaccine Dose			Negative Control Group	Adjuvant Control Group	Low-Dose Group	High-Dose Group
3 days after the last administration	Male	Epididymis	0.2658 ± 0.0367	0.2576 ± 0.0251	0.2601 ± 0.0283	0.2483 ± 0.0304
		Testis	0.6824 ± 0.0537	0.6268 ± 0.0467 *	0.6158 ± 0.0307 **	0.6315 ± 0.0390 *
		Adrenal gland	0.0096 ± 0.0023	0.0090 ± 0.0014	0.0096 ± 0.0014	0.0092 ± 0.0017
		Kidney	0.5458 ± 0.0486	0.5309 ± 0.0307	0.5248 ± 0.0453	0.5523 ± 0.0326
		Spleen	0.1523 ± 0.0189	0.1366 ± 0.0088	0.1444 ± 0.0158	0.1651 ± 0.0303
		Liver	2.3305 ± 0.1121	2.2187 ± 0.1726	2.4060 ± 0.1891	2.2698 ± 0.1473
		Thymus	0.0725 ± 0.0194	0.0603 ± 0.0137	0.0617 ± 0.0125	0.0619 ± 0.0193
		Heart	0.3378 ± 0.0348	0.3249 ± 0.0280	0.3529 ± 0.0531	0.3212 ± 0.0312
		Lung	0.3062 ± 0.0227	0.2983 ± 0.0188	0.3251 ± 0.0499	0.3198 ± 0.0167
		Brain	0.4025 ± 0.0385	0.4119 ± 0.0280	0.3869 ± 0.0401	0.3967 ± 0.0427
	Female	Ovary	0.0315 ± 0.0082	0.0306 ± 0.0041	0.0267 ± 0.0044	0.0298 ± 0.0045
		Uterus	0.2139 ± 0.0769	0.1885 ± 0.0534	0.2174 ± 0.0621	0.2001 ± 0.0632
		Adrenal gland	0.0177 ± 0.0049	0.0183 ± 0.0020	0.0179 ± 0.0025	0.0177 ± 0.0041
		Kidney	0.5516 ± 0.0259	0.5802 ± 0.0279	0.5709 ± 0.0501	0.5740 ± 0.0599
		Spleen	0.1753 ± 0.0279	0.1704 ± 0.0193	0.1706 ± 0.0221	0.1925 ± 0.0266
		Liver	2.3439 ± 0.1195	2.4132 ± 0.1315	2.3922 ± 0.1566	2.3729 ± 0.1429
		Thymus	0.1139 ± 0.0106	0.0991 ± 0.0216	0.0808 ± 0.0256 **	0.0903 ± 0.0219 *
		Heart	0.3721 ± 0.0405	0.3496 ± 0.0287	0.3569 ± 0.0364	0.3626 ± 0.0439
		Lung	0.4053 ± 0.0317	0.4093 ± 0.0277	0.4202 ± 0.0909	0.4353 ± 0.0358
		Brain	0.6161 ± 0.0499	0.6459 ± 0.0533	0.6162 ± 0.0472	0.6430 ± 0.0578
56 days after the last administration	Male	Epididymis	0.2128 ± 0.0808	0.2424 ± 0.0324	0.2430 ± 0.0709	0.2813 ± 0.0402
		Testis	0.3827 ± 0.2017	0.6005 ± 0.0379	0.5133 ± 0.2079	0.6255 ± 0.0505
		Adrenal gland	0.0103 ± 0.0028	0.0091 ± 0.0022	0.0085 ± 0.0017	0.0090 ± 0.0017
		Kidney	0.5112 ± 0.0229	0.5268 ± 0.0554	0.5078 ± 0.0195	0.5448 ± 0.0225
		Spleen	0.1383 ± 0.0250	0.1380 ± 0.0229	0.1345 ± 0.0187	0.1500 ± 0.0115
		Liver	2.2710 ± 0.1207	2.3619 ± 0.4528	2.4125 ± 0.1161	2.2655 ± 0.1379
		Thymus	0.0544 ± 0.0132	0.0594 ± 0.0157	0.0413 ± 0.0073	0.0572 ± 0.0186
		Heart	0.3346 ± 0.0581	0.3084 ± 0.0175	0.3026 ± 0.0369	0.3532 ± 0.0541
		Lung	0.3247 ± 0.0412	0.3158 ± 0.0186	0.2964 ± 0.0127	0.3189 ± 0.0276
		Brain	0.3613 ± 0.0369	0.3764 ± 0.0193	0.3539 ± 0.0178	0.4100 ± 0.0312 *
	Female	Ovary	0.0253 ± 0.0025	0.0241 ± 0.0099	0.0245 ± 0.0067	0.0215 ± 0.0050
		Uterus	0.1806 ± 0.0556	0.2303 ± 0.0542	0.1968 ± 0.0385	0.2182 ± 0.0470
		Adrenal gland	0.0180 ± 0.0030	0.0198 ± 0.0040	0.0163 ± 0.0042	0.0151 ± 0.0036
		Kidney	0.5685 ± 0.0577	0.6254 ± 0.0641	0.5547 ± 0.0283	0.5629 ± 0.0524
		Spleen	0.1531 ± 0.0067	0.1780 ± 0.0148 *	0.1568 ± 0.0194	0.1793 ± 0.0145 *
		Liver	2.2804 ± 0.1794	2.5347 ± 0.2888	2.4259 ± 0.1219	2.3602 ± 0.0854
		Thymus	0.0727 ± 0.0246	0.0776 ± 0.0122	0.0758 ± 0.0284	0.0764 ± 0.0132
		Heart	0.3088 ± 0.0235	0.3502 ± 0.0391	0.3397 ± 0.0228	0.3164 ± 0.0110
		Lung	0.3926 ± 0.0495	0.4546 ± 0.0334	0.4189 ± 0.0849	0.4111 ± 0.0196
		Brain	0.5731 ± 0.0818	0.6218 ± 0.0488	0.5853 ± 0.0688	0.6010 ± 0.0402

All groups compared with the negative control group. * *p* < 0.05; ** *p* < 0.01. The numbers in the table represent the mean ± SD.

## Data Availability

The data generated and analyzed during the current study are available from the corresponding author upon reasonable request.

## Supplementary Materials

The following supporting information can be downloaded at: https://www.mdpi.com/article/10.3390/vaccines14010029/s1, Supplement Table S1. Statistical data of animal body weight (MEAN ± SD, g); Supplement Table S2. Statistical data of food intake of experimental animals, (MEAN ± SD, g/d/rat); Supplement Table S3. Animal ophthalmic examination data; Supplement Table S4. Statistical data of urinalysis; Supplement Table S5. Statistical Data of Animal Visceral Organ Wet Weights; Supplement Table S6. Statistical data of animal cytokine assays; Supplement Table S7. Antibody positive rate after each dose of vaccination.
