# Supplementary material for: Preclinical Study on Sabin Strain-Based DTaP-sIPV/Hib Pentavalent Vaccine: Evaluation of Repeated-Dose Toxicity and Immunogenicity
Source: Vaccines (Basel). 2025 Dec 25;14(1):29. doi: 10.3390/vaccines14010029 (PMC12846469; doi:10.3390/vaccines14010029)
Supplement: Supplementary file 1 [file vaccines-14-00029-s001.zip › vaccines-4024694-supplementary.pdf]

Supplement Table S1.Statistical data of animal body weight (MEAN ± SD, g)

Number of doses	Time after each vaccine dose	Negative control group		Adjuvant control group		Low dose group		High dose group	
		M	F	M	F	M	F	M	F
Before immunization		223±9	188±10	223±8	188±8	222±8	188±8	223±8	188±9
First immunization	Week1	268±13	217±11	268±11	214±8	268±12	216±8	266±13	215±10
	Week2	324±22	254±18	318±17	243±10	321±17	249±15	319±20	249±15
	Week3	371±29	265±17	360±21	256±10	365±22	258±12	361±27	259±12
Second immunization	Week1	408±34	279±18	392±26	267±13	403±26	280±15	396±34	273±12
	Week2	432±39	289±22	418±30	277±14	433±29	293±20	423±38	281±17
	Week3	467±43	301±23	448±33	289±15	467±34	299±18	453±42	292±16
Third immunization	Week1	484±47	308±22	464±35	295±16	481±38	308±18	468±45	299±19
	Week2	504±51	312±27	482±36	300±22	505±41	314±22	485±49	309±23
	Week3	518±54	318±27	501±38	306±21	523±42	318±22	502±52	309±19
Fourth immunization	Week1	533±57	330±28	514±38	317±23	536±46	326±23	514±53	319±24
	Week2	550±62	338±32	529±42	321±24	551±48	334±26	531±56	325±28
	Week3	564±64	341±33	542±44	323±26	568±51	338±28	545±59	327±25
Fifth immunization(n=5)	Week1	564±69	345±38	559±51	330±22	568±32	335±34	507±32	322±8
	Week2	576±75	347±39	567±53	332±22	577±37	338±36	512±31	322±7
	Week3	587±78	352±42	577±58	340±27	584±44	344±38	522±33	325±9
	Week4	600±80	357±48	591±53	345±25	601±47	354±41	538±35	327±11
	Week5	604±79	361±52	600±54	342±26	607±46	355±46	542±34	331±14
	Week6	616±84	364±52	604±53	344±29	614±46	356±46	546±34	332±16
	Week7	622±84	362±51	611±56	349±31	618±49	361±50	549±36	336±17
	Week8	627±86	361±56	617±54	353±34	624±47	357±45	575±32	339±13

M:Male; F:Female.

Supplement Table S2.Statistical data of food intake of experimental animals,（MEAN±SD，g/d/rat）

Number of doses	Time after each vaccine dose	Negative control group		Adjuvant control group		Low dose group		High dose group	
		M	F	M	F	M	F	M	F
First immunization	Week1	27.0±1.3	22.2±3.0	25.0±2.7	20.7±2.5	26.9±1.2	20.6±1.4	26.3±1.8	21.1±1.9
	Week2	28.1±2.1	22.9±1.1	27.8±1.9	20.4±2.8	29.4±1.3	21.3±2.0	28.1±1.7	21.5±3.6
	Week3	28.1±1.9	19.6±3.7	26.9±1.1	20.4±1.3	30.2±2.4	19.8±2.5	27.0±3.7	18.2±1.1
Second immunization	Week1	26.6±1.8	21.2±0.8	25.8±1.0	19.9±1.8	26.7±1.2	20.4±2.5	25.4±1.6	20.1±1.6
	Week2	26.9±1.9	21.6±2.0	26.5±1.9	21.2±1.5	28.5±2.2	20.4±2.7	27.3±2.0	17.2±2.7**
	Week3	24.4±1.8	17.9±1.7	24.3±1.6	15.9±2.5	25.1±1.3	17.1±2.4	23.4±2.0	18.1±1.8
Third immunization	Week1	26.4±1.8	18.7±1.6	25.0±1.4	19.6±2.5	27.7±1.2	17.9±0.9	25.3±2.6	18.3±1.4
	Week2	27.4±1.8	18.9±1.6	26.9±1.7	21.9±6.3	27.3±1.1	19.4±1.6	25.8±2.0	18.6±1.7
	Week3	27.8±1.7	19.1±1.3	26.8±2.4	20.9±3.8	30.2±3.0	21.2±3.9	28.9±2.7	19.5±1.6
Fourth immunization	Week1	26.2±1.6	19.1±2.0	27.2±2.0	18.4±2.0	28.1±2.0	17.8±1.9	25.6±1.9	18.0±2.0
	Week2	27.3±2.5	19.1±3.1	24.8±2.9	19.8±1.1	27.3±1.8	18.6±1.8	26.6±1.6	18.5±1.2
	Week3	26.6±2.1	18.1±1.5	26.0±1.1	20.6±5.3	26.7±1.8	18.6±2.5	27.6±2.7	18.0±2.0
Fifth immunization(n=5)	Week1	24.1±0.7	17.0±2.4	26.7±0.1	18.8±0.7	27.1±1.4	16.4±0.6	23.3±1.3	17.6±0.0
	Week2	26.4±0.3	20.8±0.9	23.5±4.8	21.1±3.6	27.5±1.1	16.8±0.7	24.9±0	18.0±1.8
	Week3	27.4±0.2	20.6±0.3	27.3±0.7	18.5±2.2	27.9±0	16.4±4.0	26.0±0.8	18.1±1.1
	Week4	26.2±0.1	19.6±3.1	28.3±2.7	19.5±2.1	27.4±2.5	18.6±0.9	25.0±1.8	16.1±0
	Week5	35.1±13.2	34.8±23.8	27.9±3.3	18.5±2.7	28.5±1.1	18.3±3.3	27.4±1.9	20.2±3.6
	Week6	25.6±1.4	18.7±0.2	27.4±0.2	20.0±3.3	27.5±0.1	16.2±1.0	26.3±0.5	22.8±0
	Week7	27.4±1.1	16.4±4.1	26.8±0.2	19.5±1.0	27.3±0.9	18.1±4.7	26.5±0.6	17.1±2.1
	Week8	29.3±0.1	18.7±2.1	29.6±1.6	22.3±2.3	30.4±1.7	17.9±2.5	27.6±2.6	17.2±4.5

All group compared with the negative control group,** $P<0.01$.

M:Male; F:Female.

Supplement Table S3. Animal ophthalmic examination data

Time after each vaccine dose		Negative control group Unremarkable/Total	Adjuvant control group Unremarkable/Total	Low dose group Unremarkable/Total	High dose group Unremarkable/Total
2 days after the last administration	Male	10/10	10/10	10/10	10/10
	Female	10/10	10/10	10/10	10/10
27 days after the last administration	Male	5/5	5/5	5/5	5/5
	Female	5/5	5/5	5/5	5/5

Supplement Table S4. Statistical data of urinalysis

detection object	Time after last vaccine dose	Value	Negative control group		Adjuvant control group		Low dose group		High dose group	
			M	F	M	F	M	F	M	F
Leukocytes (LEU)	Day2	0	9/10	10/10	8/10	10/10	8/10	10/10	10/10	10/10
		15	1/10		2/10					
		70					2/10			
	Day55	0	5/5	5/5	4/5	5/5	4/5	5/5	1/5	5/5
		15			1/5		1/5		4/5	
Ketones (KET)	Day2	0	2/10	10/10	2/10	10/10	5/10	10/10	6/10	10/10
		0.5	8/10		6/10		3/10		3/10	
		1.5			2/10		2/10		1/10	
	Day55	0	3/5	5/5	4/5	4/5	1/5	4/5		4/5
		0.5	2/5			1/5	1/5	1/5		1/5
		1.5			1/5		3/5		4/5	
		4							1/5	
Nitrites (NIT)	Day2	Neg	10/10	10/10	10/10	10/10	10/10	10/10	10/10	10/10
	Day55	Neg	5/5	3/5	5/5	2/5	5/5	3/5	5/5	4/5
		Pos		2/5		3/5		2/5		1/5
Urobilinogen (URO)	Day2	Normal	10/10	10/10	9/10	10/10	8/10	10/10	10/10	10/10
		33			1/10		2/10			
	Day55	Normal	5/5	5/5	4/5	5/5	3/5	4/5		4/5
		33			1/5		2/5	1/5	5/5	1/5
Bilirubin (BIL)	Day2	0	9/10	10/10	9/10	10/10	9/10	10/10	10/10	10/10
		8.6	1/10		1/10		1/10			
	Day55	0	5/5	5/5	4/5	5/5	3/5	5/5	1/5	4/5
		8.6			1/5		2/5		4/5	1/5

Protein (PRO)	Day2	0		9/10		10/10		10/10	2/10	10/10
		0.15	4/10	1/10	4/10		5/10		3/10	
		0.3	5/10		2/10		2/10		4/10	
		1.0	1/10		4/10		3/10		1/10	
	Day55	0		4/5		4/5		4/5		3/5
		0.15	4/5	1/5	3/5	1/5	1/5			1/5
		0.3	1/5				2/5			
		1.0			2/5		2/5	1/5	5/5	
		>=3.0								1/5
Glucose (GLU)	Day2	0	10/10	10/10	10/10	10/10	10/10	10/10	10/10	10/10
	Day55	0	5/5	5/5	5/5	5/5	5/5	5/5	5/5	5/5
Specific Gravity (SG)	Day2	1.005			1/10	1/10				
		1.010	1/10		6/10	4/10	3/10	2/10	1/10	2/10
		1.015	6/10	7/10	3/10	5/10	7/10	7/10	8/10	8/10
		1.020	2/10	3/10				1/10	1/10	
		1.025	1/10							
	Day55	1.005					1/5			1/5
		1.010							1/5	1/5
		1.015	5/5	4/5	3/5		1/5	1/5	1/5	1/5
		1.020		1/5	2/5	5/5	3/5	3/5	1/5	2/5
		1.025						1/5		
Occult Blood (BLD)	Day2	0	10/10	10/10	10/10	10/10	10/10	7/10	10/10	8/10
		10						1/10		1/10
		80						1/10		1/10
		200						1/10		
	Day55	0	5/5	5/5	5/5	3/5	5/5	5/5	5/5	5/5

		10				2/5				
	Day2	6.5	2/10	1/10						
		7.0	1/10			1/10				1/10
		7.5	2/10	4/10		1/10	4/10	3/10	6/10	1/10
		8.0	2/10	2/10	8/10	6/10	2/10	5/10	4/10	4/10
pH		8.5	3/10	3/10	2/10	2/10	4/10	2/10		4/10
	Day55	7.0		1/5			3/5			
		7.5			1/5	2/5		3/5	2/5	2/5
		8.0	5/5	1/5	4/5	3/5	2/5	2/5	3/5	
		8.5		3/5						3/5
Vitamin C (Vit C)	Day2	0	10/10	10/10	10/10	10/10	10/10	10/10	10/10	10/10
	Day55	0	5/5	5/5	5/5	5/5	5/5	5/5	5/5	5/5
Color	Day2	light yellow	10/10	10/10	10/10	10/10	10/10	10/10	10/10	10/10
	Day55	light yellow	5/5	5/5	5/5	5/5	5/5	5/5	5/5	5/5
Turbidity	Day2	clear	10/10	10/10	10/10	10/10	10/10	10/10	10/10	10/10
	Day55	clear	5/5	5/5	5/5	5/5	5/5	5/5	5/5	5/5

M:Male; F:Female.

Supplement Table S5. Statistical Data of Animal Visceral Organ Wet Weights

Time after each vaccine dose			Negative control group	Adjuvant control group	Low dose group	High dose group
3 days after the last administration	Male	Epididymis	1.4220±0.1393	1.3351±0.1269	1.4240±0.2141	1.3379±0.1308
		Testis	3.6581±0.1332	3.2615±0.3853	3.3715±0.3788	3.4260±0.4171
		Adrenal gland	0.0511±0.0114	0.0465±0.0080	0.0523±0.0098	0.0493±0.0075
		Kidney	2.9344±0.3011	2.7529±0.1872	2.8687±0.3418	3.0043±0.4294
		Spleen	0.8192±0.1051	0.7096±0.0676	0.7960±0.1536	0.8963±0.1999
		Liver	12.5829±1.4506	11.5402±1.4022	13.2364±2.1543	12.3655±1.8655
		Thymus	0.3936±0.1118	0.3106±0.0624	0.3377±0.0793	0.3334±0.0973
		Heart	1.8157±0.1933	1.6894±0.2074	1.9270±0.3072	1.7381±0.2079
		Lung	1.6462±0.1389	1.5476±0.1219	1.7617±0.1725	1.7350±0.1969
		Brain	2.1554±0.1065	2.1317±0.0826	2.1403±0.1028	2.1380±0.1630
	Female	Ovary	0.1012±0.0275	0.0943±0.0154	0.0867±0.0138	0.0940±0.0145
		Uterus	0.6801±0.2149	0.5779±0.1555	0.7072±0.2093	0.6232±0.1747
		Adrenal gland	0.0570±0.0169	0.0563±0.0073	0.0582±0.0074	0.0554±0.0117
		Kidney	1.7734±0.1287	1.7862±0.1714	1.8517±0.0925	1.8038±0.1280
		Spleen	0.5629±0.0938	0.5229±0.0600	0.5558±0.0805	0.6084±0.0970
		Liver	7.5648±0.9332	7.4317±0.7720	7.7712±0.4185	7.4809±0.5553
		Thymus	0.3670±0.0462	0.3034±0.0658	0.2661±0.0939**	0.2851±0.0700*
		Heart	1.1996±0.1707	1.0748±0.1123	1.1608±0.1246	1.1487±0.1903
		Lung	1.3032±0.1269	1.2540±0.0410	1.3730±0.3410	1.3711±0.1115
		Brain	1.9734±0.0735	1.9767±0.0554	1.9991±0.0807	2.0187±0.0610
56 days after the last administration	Male	Epididymis	1.2814±0.4934	1.4591±0.2498	1.4783±0.4519	1.5095±0.1507
		Testis	2.3196±1.2530	3.6028±0.3416	3.1016±1.2169	3.3629±0.0912

	Adrenal gland	0.0610±0.0124	0.0541±0.0117	0.0520±0.0130	0.0485±0.0095
	Kidney	3.1034±0.4668	3.1792±0.5750	3.0840±0.1773	2.9445±0.2769
	Spleen	0.8351±0.1705	0.8245±0.1153	0.8175±0.1238	0.8098±0.0810
	Liver	13.7574±1.8453	14.3417±3.9533	14.6509±0.9341	12.2266±1.0694
	Thymus	0.3223±0.0448	0.3538±0.0789	0.2516±0.0485	0.3058±0.0948
	Heart	2.0031±0.2355	1.8538±0.2114	1.8400±0.2617	1.9089±0.3475
	Lung	1.9457±0.1453	1.8961±0.1937	1.8010±0.1244	1.7197±0.1683
	Brain	2.1713±0.1752	2.2531±0.0814	2.1493±0.1426	2.2058±0.1000
Female	Ovary	0.0882±0.0168	0.0795±0.0280	0.0819±0.0154	0.0704±0.0171
	Uterus	0.6094±0.1162	0.7870±0.2490	0.6763±0.1636	0.7116±0.1537
	Adrenal gland	0.0633±0.0147	0.0672±0.0179	0.0552±0.0133	0.0494±0.0130
	Kidney	1.9670±0.2308	2.1108±0.3153	1.8911±0.2014	1.8308±0.1194
	Spleen	0.5333±0.0742	0.5986±0.0653	0.5299±0.0292	0.5837±0.0389
	Liver	7.8914±0.7730	8.5807±1.5749	8.2958±1.1528	7.6925±0.3357
	Thymus	0.2557±0.0937	0.2635±0.0598	0.2514±0.0777	0.2500±0.0503
	Heart	1.0708±0.1272	1.1812±0.1790	1.1550±0.0959	1.0317±0.0551
	Lung	1.3521±0.1179	1.5269±0.1204	1.4052±0.1342	1.3387±0.0234
	Brain	1.9653±0.0433	2.0846±0.1036	1.9775±0.0535	1.9564±0.0897

Supplement Table S6. Statistical data of animal cytokine assays

Time after each vaccine dose			Negative control group		Adjuvant control group		Low dose group		High dose group	
			n	MEAN±SD，pg/ml	n	MEAN±SD，pg/ml	n	MEAN±SD，pg/ml	n	MEAN±SD，pg/ml
3 days after the last administration	Male	IFN-γ	10	40.96±12.56	10	36.99±13.19	10	31.46±9.24	10	43.59±16.45
		TNF-α	4	13.29±4.45	5	2.99±1.98*	4	17.44±17.18	3	25.57±7.30
		IL-2	0	/	0	/	0	/	1	100.2
	Female	IFN-γ	10	129.53±79.16	10	92.60±12.05	10	98.75±27.24	10	119.37±47.85
		TNF-α	10	108.18±10.07	10	110.42±18.46	10	107.93±24.93	10	114.82±31.45
		IL-2	5	25.36±18.51	3	3.95±2.07	2	14.91±17.35	4	27.91±24.70
56 days after the last administration	Male	IFN-γ	1	5.22	0	/	0	/	0	/
		TNF-α	5	19.31±19.17	5	21.07±15.61	5	19.41±17.25	5	21.78±21.50
		IL-2	0	/	0	/	0	/	0	/
	Female	IFN-γ	0	/	0	/	0	/	0	/
		TNF-α	4	17.30±9.95	4	44.38±40.23	5	20.62±13.58	5	22.80±21.49
		IL-2	0	/	0	/	0	/	0	/

All group compared with the negative control group,*P<0.05

Supplement Table S7. Antibody positive rate after each dose of vaccination

Types of antibody	Time after each vaccine dose	Negative control group		Low dose group		High dose group	
		n/10	%	n/10	%	n/10	%
DT	Before immunization	0/10	0%	0/10	0%	0/10	0%
	21 days after the first immunization	0/10	0%	10/10	100%	10/10	100%
	21 days after the second immunization	0/10	0%	10/10	100%	10/10	100%
	21 days after the third immunization	0/10	0%	10/10	100%	10/10	100%
	21 days after the fourth immunization	0/10	0%	10/10	100%	10/10	100%
	21 days after the fifth immunization	0/10	0%	10/10	100%	10/10	100%
TT	Before immunization	0/10	0%	0/10	0%	0/10	0%
	21 days after the first immunization	0/10	0%	10/10	100%	10/10	100%
	21 days after the second immunization	0/10	0%	10/10	100%	10/10	100%
	21 days after the third immunization	0/10	0%	10/10	100%	10/10	100%
	21 days after the fourth immunization	0/10	0%	10/10	100%	10/10	100%
	21 days after the fifth immunization	0/10	0%	10/10	100%	10/10	100%
PT	Before immunization	0/10	0%	0/10	0%	0/10	0%
	21 days after the first immunization	0/10	0%	10/10	100%	10/10	100%
	21 days after the second immunization	0/10	0%	10/10	100%	10/10	100%
	21 days after the third immunization	0/10	0%	10/10	100%	10/10	100%
	21 days after the fourth immunization	0/10	0%	10/10	100%	10/10	100%
	21 days after the fifth immunization	0/10	0%	10/10	100%	10/10	100%
FHA	Before immunization	0/10	0%	0/10	0%	0/10	0%
	21 days after the first immunization	0/10	0%	10/10	100%	10/10	100%

	21 days after the second immunization	0/10	0%	10/10	100%	10/10	100%
	21 days after the third immunization	0/10	0%	10/10	100%	10/10	100%
	21 days after the fourth immunization	0/10	0%	10/10	100%	10/10	100%
	21 days after the fifth immunization	0/10	0%	10/10	100%	10/10	100%
PRN	Before immunization	0/10	0%	0/10	0%	0/10	0%
	21 days after the first immunization	0/10	0%	10/10	100%	10/10	100%
	21 days after the second immunization	0/10	0%	10/10	100%	10/10	100%
	21 days after the third immunization	0/10	0%	10/10	100%	10/10	100%
	21 days after the fourth immunization	0/10	0%	10/10	100%	10/10	100%
	21 days after the fifth immunization	0/10	0%	10/10	100%	10/10	100%
PRP	Before immunization	0/10	0%	0/10	0%	0/10	0%
	21 days after the first immunization	0/10	0%	10/10	100%	10/10	100%
	21 days after the second immunization	0/10	0%	10/10	100%	10/10	100%
	21 days after the third immunization	0/10	0%	10/10	100%	10/10	100%
	21 days after the fourth immunization	0/10	0%	10/10	100%	10/10	100%
	21 days after the fifth immunization	0/10	0%	10/10	100%	10/10	100%
PV1	Before immunization	0/10	0%	0/10	0%	0/10	0%
	21 days after the first immunization	0/10	0%	5/10	50%	6/10	60%
	21 days after the second immunization	0/10	0%	10/10	100%	10/10	100%
	21 days after the third immunization	0/10	0%	10/10	100%	10/10	100%
	21 days after the fourth immunization	0/10	0%	10/10	100%	10/10	100%
	21 days after the fifth immunization	0/10	0%	10/10	100%	10/10	100%

PV2	Before immunization	0/10	0%	0/10	0%	0/10	0%
	21 days after the first immunization	0/10	0%	5/10	50%*	3/10	30%
	21 days after the second immunization	0/10	0%	9/10	90%	10/10	100%
	21 days after the third immunization	0/10	0%	10/10	100%	10/10	100%
	21 days after the fourth immunization	0/10	0%	10/10	100%	10/10	100%
	21 days after the fifth immunization	0/10	0%	10/10	100%	10/10	100%
PV3	Before immunization	0/10	0%	0/10	0%	0/10	0%
	21 days after the first immunization	0/10	0%	5/10	50%	7/10	70%
	21 days after the second immunization	0/10	0%	9/10	90%	10/10	100%
	21 days after the third immunization	0/10	0%	10/10	100%	10/10	100%
	21 days after the fourth immunization	0/10	0%	10/10	100%	10/10	100%
	21 days after the fifth immunization	0/10	0%	10/10	100%	10/10	100%
